# Primary histologic diagnosis using automated whole slide imaging: a validation study

**DOI:** 10.1186/1472-6890-6-4

**Published:** 2006-04-27

**Authors:** John R Gilbertson, Jonhan Ho, Leslie Anthony, Drazen M Jukic, Yukako Yagi, Anil V Parwani

**Affiliations:** 1 Center for Pathology Informatics, University of Pittsburgh Medical Center, Pittsburgh, PA, 15232, USA; 2 Center for Biomedical Informatics, University of Pittsburgh, Pittsburgh, PA, 15213, USA; 3 Department of Pathology, University of Pittsburgh School of Medicine, UPMC Presbyterian/Shadyside, Pittsburgh, PA, 15232, USA; 4 Department of Dermatology, University of Pittsburgh School of Medicine, UPMC Presbyterian/Shadyside, Pittsburgh, PA, 15232, USA; 5 Benedum Center for Oncology Informatics, University of Pittsburgh Medical Center, Pittsburgh, PA, 15232, USA

## Abstract

**Background:**

Only prototypes 5 years ago, high-speed, automated whole slide imaging (WSI) systems (also called digital slide systems, virtual microscopes or wide field imagers) are becoming increasingly capable and robust. Modern devices can capture a slide in 5 minutes at spatial sampling periods of less than 0.5 micron/pixel. The capacity to rapidly digitize large numbers of slides should eventually have a profound, positive impact on pathology. It is important, however, that pathologists validate these systems during development, not only to identify their limitations but to guide their evolution.

**Methods:**

Three pathologists fully signed out 25 cases representing 31 parts. The laboratory information system was used to simulate real-world sign-out conditions including entering a full diagnostic field and comment (when appropriate) and ordering special stains and recuts. For each case, discrepancies between diagnoses were documented by committee and a "consensus" report was formed and then compared with the microscope-based, sign-out report from the clinical archive.

**Results:**

In 17 of 25 cases there were no discrepancies between the individual study pathologist reports. In 8 of the remaining cases, there were 12 discrepancies, including 3 in which image quality could be at least partially implicated. When the WSI consensus diagnoses were compared with the original sign-out diagnoses, no significant discrepancies were found. Full text of the pathologist reports, the WSI consensus diagnoses, and the original sign-out diagnoses are available as an attachment to this publication.

**Conclusion:**

The results indicated that the image information contained in current whole slide images is sufficient for pathologists to make reliable diagnostic decisions and compose complex diagnostic reports. This is a very positive result; however, this does not mean that WSI is as good as a microscope. Virtually every slide had focal areas in which image quality (focus and dynamic range) was less than perfect. In some cases, there was evidence of over-compression and regions made "soft" by less than perfect focus. We expect systems will continue to get better, image quality and speed will continue to improve, but that further validation studies will be needed to guide development of this promising technology.

## Background

Automated, high-speed, high-resolution whole side imaging (WSI) robots, introduced as prototypes only a few years ago, are becoming increasingly robust and capable. Current devices can automatically image several hundred slides per day and these volumes will only increase in the future. With time, this technology should have a significant and beneficial impact on pathology practice as it will enable pathologists to better apply computational power and network connectivity to all aspects of their practice, not just reporting and accessioning. Though several years off, integrated digitization (gross, slides and text) could streamline workflows, increase productivity, and allow new and unforeseen capabilities such as computer-aided diagnosis (CAD). Despite these promises, however, there exists only a relatively small number of papers describing automated WSI in education and research [1-6], far fewer directly comparing automated WSI with traditional microscope examination in diagnosis [7-11], and virtually none describing automated WSI in clinical practice. A good review of WSI technology and applications can be found in Gu and Ogilvie [12].

Clinical (diagnostic) validation of WSI systems is extremely important because image capture is automated, focusing mechanisms are novel, and the size of the datasets they generate mandates the use of lossy compression. Validation studies are also an important way for pathologists to guide the development of the technology. There is little question that digitization itself is not a barrier to pathology diagnosis; for many years pathology practices have provided remote diagnostic services using robotic real-time, near real-time and even static image-based telepathology. For example, Dr. Bruce Dunn and his colleagues at the Milwaukee Veterans Administration reported thousands of successful diagnostic robotic telepathology sessions with a remote hospital in Iron Mountain, MI [13]; Dr. Keith Kaplan established an active robotic telepathology service that now supports 22 army facilities [14]; and Drs. Marida Minervini and Jake Demetrius and their colleagues in Pittsburgh used a static telepathology system to support transplant pathology at a hospital in Palermo Italy [15]. In these traditional telepathology applications, however, pathologists directly control the image formation (focus) and capture process. This is not the case with high-speed WSI systems in which image formation and capture are automated.

Given the large number of slides generated by pathology laboratories, one of the most essential features of a clinical WSI system is slide capture speed (or throughput). Fortunately, modern WSI devices are becoming very fast, with current devices ranging from 2 to 8 minutes per slide. To achieve this speed, systems employ a unique focusing strategy. While some WSI systems can be set to auto-focus at every point on the slide [16-18], this is a time consuming process. Systems designed for faster speeds tend to pre-focus on a limited number of points and use proprietary algorithms to calculate an "in focus surface" above the slide such that, if the objective lens follows that surface during capture, the resulting image should be in focus. While engineers are developing increasingly sophisticated and successful algorithms, and increasingly capable stages for implementing these algorithms, perfect focus is rarely achieved over an entire slide. This is largely because tissue sections are not planar, especially compared to the depth of field produced by high numerical aperture 20 × or 40 × objectives. Some researchers have suggested that vendors decrease the numerical aperture of their lenses or stop down the condenser – effectively trading lower maximal optical resolution for a deeper depth of field [19]. However, achieving perfect focus across an entire slide remains a major challenge for modern high-speed WSI systems and a potential limitation to their clinical use. Therefore, the high degree of diagnostic capability expected from a traditional, microscope-based pathology examination and reported for robotic telepathology systems (vide infra) [13-15] cannot be assumed in automated WSI and must be validated in controlled studies.

In a WSI system, the optics form an image, the focusing system places the image precisely on the sensor (usually a CCD) and the CCD/camera samples that image. Captured images are generally subjected to compression and are viewed on a wide range of monitors. While the quality of focus is often considered the most critical aspect of image quality, these other components (and their interactions) are also important. In actual practice, if WSI systems have quality optics with sufficient numerical aperture (as found in most current devices), and if precise focus is achieved (vide supra), then the quality of the captured image will be determined by the quality of the image sampling. Sampling is limited by the spatial sampling period ("pixel resolution") [20] and dynamic range of the sensor. Spatial sampling period is calculated by dividing the size of a pixel by the primary optical magnification of the system. For example, a CCD with 6.6 micron pixels and 20 × objective lens gives a sampling period of 333 nanometers. While manufacturers (and users) often tout spatial sampling period as a proxy for image quality, in reality captured image quality is a function of many parameters (including optical resolution, focus, spatial sampling period and dynamic range) and relationships between these variables are complex issues well beyond the scope of this paper [21]. To improve image quality, for example, one may choose to decrease (improve) the spatial sampling period by increasing the magnification or decreasing the pixel size. However, these changes can have unintended consequences: increasing magnification often increases numeric aperture which could affect focus while smaller pixels could decrease dynamic range and actually decrease image quality. The point is that relationships between image quality, optical resolution, sampling period, dynamic range, etc. are complex and ideal combinations cannot be easily predicted. Again, careful study, testing and validation are needed to guide development and practice.

Another important parameter that impacts image quality is the use of compression. The effect of compression on traditional static and dynamic telepathology has been well studied [22]. A typical WSI system with a sampling period of 0.33 um/pixel generates 900 million image pixels per square centimeter of tissue section. At 24 bit color, this is 2.7 GB of data per square centimeter. Therefore, all high-speed WSI devices use lossy compression as a matter of course. Although it is our experience that "reasonable" compression, especially using JPG or JG2000 algorithms, provides excellent image quality with limited artifact, the effect of compression has not been carefully studied on WSI diagnosis and its clinical impact (or lack there of) should not be taken on faith.

These considerations and many others make it clear that, for automated, high-speed WSI to reach its potential in pathology practice, it must be continuously evaluated and validated by the pathology community. In this paper we present a retrospective validation study based on 25 randomly selected genitourinary and dermatopathology cases. Our goal was to determine if pathologists could effectively sign-out complex cases (full final diagnosis field with comment) exclusively through whole slide images captured by a modern imaging robot. We also wanted to establish a level of baseline diagnostic capability for pathologists evaluating WSI for their practices and to identify problem areas that could be addressed by future focused studies.

## Methods

### Study design and participants

This was a retrospective, comparative study evaluating the use of automated, high-speed WSI in a routine clinical activity: primary diagnosis sign-out. The study took place in the Division of Anatomic Pathology at University of Pittsburgh Medical Center, Shadyside Hospital, a 486-bed academic tertiary care hospital. The division had nine faculty (sign-out) pathologists. They were all teaching faculty of the University of Pittsburgh with between 2 – 30 years of practice experience (mean = 12.8), board certifications, and fellowship training in at least one area. Three had specific formal training in GU pathology, three others consider GU pathology an area of interest or strength, and two had specific training in dermatopathology. Faculty offices and sign-out rooms were equipped with Olympus BX45 microscopes. The objective lenses were Plan Apo (magnifications range from 2 – 100 ×) and the scopes were professionally calibrated twice a year. Cases were signed out with the assistance of residents and fellows.

Three anatomic pathologists from the division with a special interest in this research volunteered participation as subjects in this study. The pathologists had at least two years of experience using WSI for education and case presentation and had participated in at least one prior WSI validation study. They included a board certified pathologist/dermatopathologist with an interest in genitourinary pathology and five years of practice, a board certified pathologist with a year of formal training in genitourinary pathology and two years of practice, and a pathology fellow in his fifth year of post-graduate training.

In addition to the study pathologists, a team of individuals with diverse and distinct backgrounds was required for this study, including:

• A project manager and a principal faculty investigator (a pathologist), responsible for the design, management, integration, and overall execution of the study;

• Evaluators with the University of Pittsburgh Center for Biomedical Informatics, responsible for protocol development, IRB approvals, data management, and focus group (de-briefing) interviews;

• The Quality Assurance Division of the Department of Pathology, responsible for independent case selection;

• A laboratory information system (LIS) team, responsible for "recreating" the study cases for sign-out by the study pathologists through the LIS;

• The histology laboratory, responsible for recuts or special stains requested by the pathologists during the study;

• An honest broker, responsible for de-identifying case material; and

• An imaging team responsible for whole slide image capture, storage, and presentation.

### Procedures and apparatus

The study was structured to enable study pathologists to independently sign-out the same 25 archived cases as if each case were "new" and each study pathologist was the responsible case pathologist. The LIS and clinical standard operating procedures for Shadyside Hospital were used throughout the study. To this end, two important aspects of the study workflow had to be implemented:

1. Study pathologists were required to use the LIS to receive and sign-out cases as well as to order special stains and recuts.

2. Consistent with new cases, study pathologists initially received only digitized images of the slides that the original case pathologists initially received. Study pathologists could request special stains and recuts as needed. When a stain or recut was requested, if the stain had been ordered and prepared for the original case pathologist, the existing slide was imaged and presented to the study pathologist. If the original case pathologist had not ordered the requested stain or recut, the histology lab cut the block and produced a new stained slide which was then imaged and presented to the study pathologist.

The only differences between the study sign-out procedure and the "original" or "real" sign-out procedure were that study pathologists worked entirely through whole slide images to diagnose the cases, they were not assisted by residents, and they did not have access to the transcription service.

Cases were limited to genitourinary pathology and dermatopathology based on the interests of study pathologists. Case selection was performed by the Department of Pathology Quality Assurance Division and was not influenced by study personnel. Cases were selected randomly using an existing computer program in the LIS normally used to select cases for quality assurance studies. The initial selection process resulted in five vasectomy cases, three of which (case numbers 32, 34 and 41) were removed before the study began and were replaced by other randomly chosen cases. Once Quality Assurance personnel assured that study pathologists were not previously associated with the selected cases, the case list was presented to the LIS team.

The LIS team "recreated" each case so that it was presented to the study pathologists in the same manner in which it had been presented to the original case pathologist. This involved removing microscope descriptions, final diagnoses, diagnostic codes, records of special stains and recuts, consultations, etc., and retaining case data in demographic, clinical history and gross description fields.

Histology pulled all the sides for each case. The honest broker "de-identified" the cases by eliminating all patient, pathologist, accession and clinical identifiers (as well any other HIPAA safe harbor data) from the reports. Study numbers were assigned to each case and new (opaque) slide labels with bar codes (for use in imaging), study numbers, block and slide numbers and stain information, were placed over the original labels. The project manager and principal investigator (a pathologist) determined which slides were "original" to the case and which represented special stains and recuts ordered by the clinical case pathologist. Original slides were sent for imaging (vide infra) while special stains and recuts were held by the project manager and imaged only when and if requested by a study pathologist (vide supra).

The study pathologists worked through the cases independently, ordering special stains and recuts as needed. Pathologists could not request that a slide be rescanned. They did not have access to the original pathology report or the glass slides and they did not discuss the cases between them. LIS logs were examined to insure that study pathologist did not attempt to use LIS text searches to "find" original archived sign-out reports. Consultation with other (non-study) pathologists was not discouraged, but such "intra-departmental consultation" was, in fact, very limited. For each case, each pathologist wrote a complete diagnostic line, including a diagnostic comment if appropriate.

After all cases were completed, study pathologists, evaluators, the project manager and the principal investigator met as a group to discuss results. The post-diagnostic discussion had two parts for each case:

1. Reports from the individual study pathologists were compared. When there were discrepancies, the pathologists discussed the case and examined the whole slide images as a group to determine the nature and extent of disagreement and the root of the discrepancy. Pathologists then reached a "WSI consensus". The principal investigator wrote down this consensus diagnosis before moving to the second part of the evaluation.

2. Once a consensus diagnostic line was agreed upon and documented, the evaluation team, for the first time, presented the original, de-identified sign-out report to the study pathologists and principal investigator. The team then determined the extent of agreement between the WSI consensus diagnosis and the microscopic-based diagnosis of the original case pathologist. Discussions involved detailed examination of the original pathology report, the digital slides and the glass slides under a multi-headed microscope.

The process was performed as if it were a strict quality assurance protocol.

### Case evaluation form and post study focus group

During the study, the pathologists were asked to fill out an evaluation form for each case. The data collected included subjective ratings of image quality (for each slide), case complexity, diagnostic confidence and system performance.

At the conclusion of the study, the evaluation team conducted a focus group interview with study pathologists. Following a round-robin format, participants were asked to contribute their thoughts on the key take-away lessons from this project. Responses were audio-recorded and transcribed. The evaluation team analyzed transcripts and identified emerging themes.

### WSI

Automated, whole slide image capture was performed on an Aperio T2 slide scanner (Aperio Technologies, Vista CA) outfitted with a Nikon Plan Fluor 20 ×, 0.7 Numerical Aperture Objective Lens and Basler L301"trilinear array" line scan camera. The system's spatial sampling period (the area of tissue section subtended by a pixel) was approximately 0.46 microns/pixel. The system ran in automated batch mode with automated focus and tissue finding. Images were compressed during the capture process in a multi-layered JPG2000 format using a Matrox Morphis compression board (Matrox Incorporated, Montreal Canada) with a quality setting of 30 resulting in file sizes ranging between 10 MB to 300 MB. The image server was a Dell machine equipped with dual Xeon processors, 4 GB of RAM, and 1 TB of hard drive storage running Microsoft Windows Server 2000, IIS and Aperio Image Server software version 5.6.

A total of 142 "original" slides were imaged at the beginning of the project. These images were grouped by case and copied into three folders, one for each study pathologist. During the study, 68 additional slides were imaged representing recuts or special stains ordered by individual study pathologists. These images were placed in the ordering pathologist's folder. Average slide capture speed was approximately 6 minutes. Following automated image capture, the imaging technician quickly examined each image at low power for general image quality. On the basis of this examination, 10 of 210 slides showed evidence that "tissue finding" function had failed and that significant areas of the tissue had not been imaged (this usually involved immunohistochemistry slides with light counter staining). In those cases, the technician manually adjusted the imaging window and let the system re-imaged the slide. There were no other technical problems with image capture.

Pathologists viewed the whole slide images through network connections on remote workstations located in their hospital offices or at their homes. Intra-hospital connections used a 100 Mbps switched Ethernet line, while off-site connections were physically limited to ISP connection speeds (approximately 1.5 Mbps). In virtually all cases, workstations and the server were on different sub-networks. Workstations (and monitors) used to view the whole slide images varied greatly, ranging from laptops to typical current-generation hospital workstations. Viewer software was ImageScope v 5.03 (Aperio Technologies, Vista CA).

## Results

### Case detail

The study was performed during a four-week period on 25 pathology cases with 31 individual parts representing 26 diagnostic biopsies, two vasectomies, a nephrectomy, a transurethral resection of the prostate, and a transurethral resection of the bladder (Table 1).

**Table 1:** Case detail

**Case**	**Part**	**Slides**	**Specimen**	**High Level Diagnosis**
31	1	1	Bladder Biopsy	Urothelial Carcinoma, High Grade
32				
33	1	4	Donor Bladder Bx	Chronic Cystitis, Moderate
34				
35	1	2	Vasectomy	Complete Excision, Bilateral
36	1	2	Vasectomy	Complete Excision, Bilateral
37	1	3	Bladder Biopsy	Urothelial Carcinoma, High Grade
38	1	3	Bladder Biopsy	Chronic Cystitis & Granulomatous Inflammation
	2	3	Bladder Biopsy	Chronic Cystitis & Granulomatous Inflammation
39	1	3	Bladder Biopsy	Urothelial Carcinoma, High Grade
40	1	3	Bladder Biopsy	Urothelial Carcinoma, High Grade
41				
42	1	9	Nephrectomy	Renal Cell Carcinoma
43	1	2	Bladder Biopsy	Chronic Cystitis & Granulomatous Inflammation
44	1	12	TURP	Benign Prostatic Tissue
45	1	9	TURB	Urothelial Carcinoma, High Grade
46	1	4	Skin Biopsy	Dermal Nevus
47	1	2	Skin Biopsy	Basal Cell Carcinoma
48	1	2	Skin Biopsy	Mycosis Fungoides
49	1	2	Skin Biopsy	Compound Nevus
50	1	3	Skin Biopsy	Basal Cell Carcinoma
	2	3	Skin Biopsy	Compound Nevus
51	1	1	Skin Biopsy	Neurofibroma
52	1	2	Skin Biopsy	Inflamed Seborrheic Keratosis
53	1	1	Skin Biopsy	Malignant Melanoma
54	1	4	Skin Biopsy	Dysplastic Nevus
55	1	4	Skin Biopsy	Compound Nevus
	2	4	Skin Biopsy	Nevus of Acral Skin
56	1	9	Prostate Biopsy	Benign Prostatic Tissue
	2	9	Prostate Biopsy	Benign Prostatic Tissue
57	1	6	Prostate Biopsy	Prostatic Adenocarcinoma, Gleason 7
	2	6	Prostate Biopsy	High Grade PIN
58	1	12	Prostate Biopsy	Prostatic Adenocarcinoma, Gleason 6
	2	12	Prostate Biopsy	Prostatic Adenocarcinoma, Gleason 6
25	31	142		

Description: Twenty-five genitourinary or dermatology cases (representing 31 individual parts) were selected randomly by the LIS. Cases 32, 34 and 41 were vasectomy specimens that were removed before the study began and replaced by more challenging cases (chosen at random). The 142 slides represented those originally cut on the case and did not include recuts and special stains ordered by the original sign-out pathologist. As part of their workup, the study pathologists ordered 68 recuts and special stains, making the total number of slides imaged in the study 210. "High Level Diagnosis" is the summary/abstraction from the first line from the original, sign–out diagnosis and can be used to judge the nature of the cases studied.

TURP = Transurethral resection of prostate

TURB = Transurethral resection of bladder tumor

As discussed in the Methods section, the study can be considered in two related parts. In the first, the three study pathologists used WSI to independently diagnose each case with a complete written diagnostic line and comment (if necessary), and the consensus committee compared those reports, documented discrepancies between them and formed a consensus WSI diagnosis that was then written down by the principal investigator. In the second part, the consensus WSI diagnosis was compared to the traditional microscope-based report of the original sign-out pathologist and any discrepancies were examined. Results for each part of the study are presented separately.

The Appendix, an additional file submitted with this paper, includes the WSI-based diagnoses of the individual study pathologists, the WSI consensus diagnoses, and the original, traditional microscope-based, sign-out diagnoses for each case. It also documents the judgments of the consensus committee in forming the WSI consensus diagnoses and the comparison of the WSI consensus diagnoses with the original sign-out reports. The results presented below can be examined further by referring to the Appendix.

### WSI concordance between study pathologists

In the first phase of the study, three pathologists signed out 25 cases representing 31 specimens (parts). This resulted in 93 independent "diagnoses" each of which included multiple (variable) diagnostic lines (e.g., Urothelial Carcinoma: Grade 2 of 3: tumor is confined to the lamina propria: Angio-lymphatic invasion is not identified). In 16 of 25 cases, representing 21 of 31 parts, there was complete agreement across the WSI-based diagnoses of the three study pathologists. In the remaining 10 parts, there were 12 diagnostic discrepancies with 2 parts having 2 discrepancies. Image quality is potentially implicated in 3 of these 12 discrepancies as discussed below.

The 12 diagnostic discrepancies between the individual WSI-based reports can be classified into four main types using data from the consensus committee (Appendix). Table 2 summarizes this information.

**Table 2:** Discrepancies between individual study pathologists' WSI-based reports

**Case**	**Part**	**Specimen**	**At Issue**	**Discrepancy Type**
37	1	Bladder Biopsy	Tumor Grade*	Type 3
38	1	Bladder Biopsy	Dysplasia v Benign	Type 4*
			Granulomatous Inflammation?	Type 2
	2	Bladder Biopsy	Granulomatous Inflammation?	Type 2
40	1	Bladder Biopsy	Tumor Grade	Type 3
			Superficial Invasion?	Type 3
45	1	Bladder Biopsy	Muscle Invasion?	Type 1
50	2	Skin Biopsy	Atypical Melanocytic Lesion: Invasion?	Type 4*
52	1	Skin Biopsy	Actinic Keratosis versus Seborrheic	Type 3
			Keratosis with HPV infection	
54	1	Bladder Biopsy	AK versus Artifact	Type 3
55	1	Skin Biopsy	Junctional versus Compound Nevus	Type 2
57	2	Prostate Bx	Atypical Glands (Cancer v HGPIN)	Type 4*

Description: There were 12 discrepancies between study pathologists' WSI-based reports. Of these, sub-optimal image quality was implicated in 4 instances (38, 50 and 57). See text for the definition of the "discrepancy types".

Type 1 discrepancies: These represent situations in which a pathologist misdiagnosed a fairly straightforward, well-imaged lesion.

**Figure 1:**
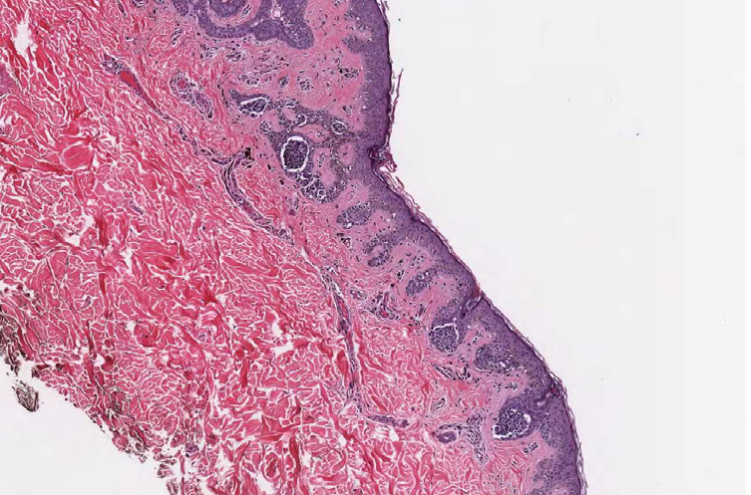
**Case 54, dysplastic nevus**. Case 54 included a dysplastic nevus and neurotized dermal nevus. Figure 1 documents the dysplastic component at medium resolution.

**Figure 2:**
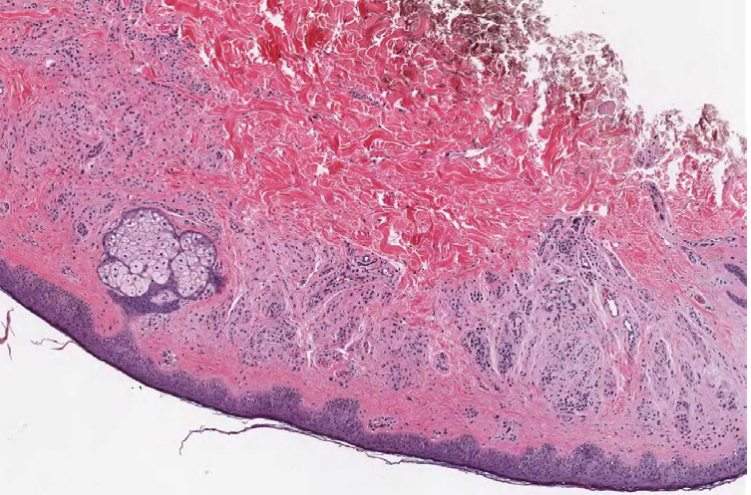
**Case 54, neurotized dermal nevus**. Case 54 included a dysplastic nevus and neurotized dermal nevus. Figure 2 documents the dermal nevus at medium resolution.

**Figure 3:**
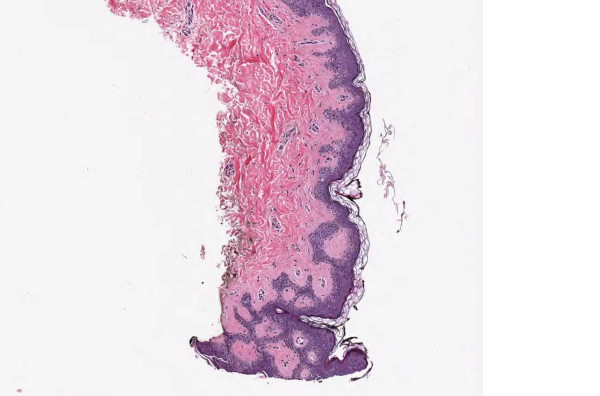
**Case 54, Tangential cut called an actinic keratosis**. Case 54 included a dysplastic nevus and neurotized dermal nevus. One pathologist also reported an AK at the edge of one of the sections. Consensus was that the "AK" was actually a tangential cut at edge of section. See Figure 4 for higher power view.

**Figure 4:**
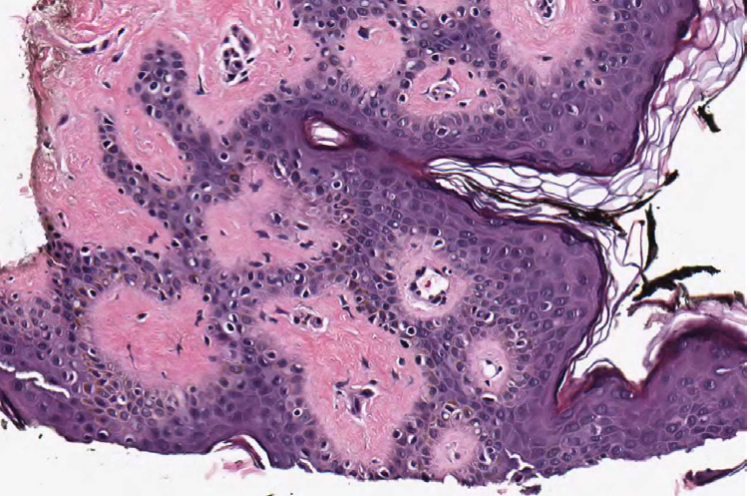
**Case 54, Tangential cut called an actinic keratosis**. Case 54 included a dysplastic nevus and neurotized dermal nevus. One pathologist also reported an AK at the edge of one of the sections. Consensus was that "AK" was actually a tangential cut at edge of section.

There were two such cases:

▪ **Case 54 **was an excision of a dysplastic nevus and neurotized dermal nevus. A pathologist reported (in addition to the diagnosis above) an actinic keratosis at the edge of the resection (Figures 1, 2, 3 and 4). At the WSI consensus committee meeting, the other pathologists convinced him that, because there was no atypia and no significant solar elastosis, the area was most likely tangential cut through a fold at the edge of the section.

**Figure 5:**
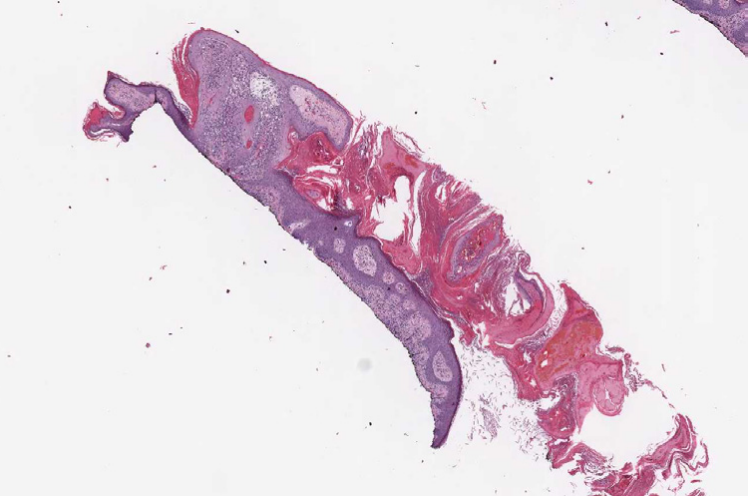
**Case 52, seborrheic keratosis**. Case 52 was a seborrheic keratosis. One study pathologist diagnosed actinic keratosis citing the mild atypia in the epithelium. Consensus was that the correct diagnosis was seborrheic keratosis. Figure 6 shows a higher power including some of the atypical cells in the epithelium.

**Figure 6:**
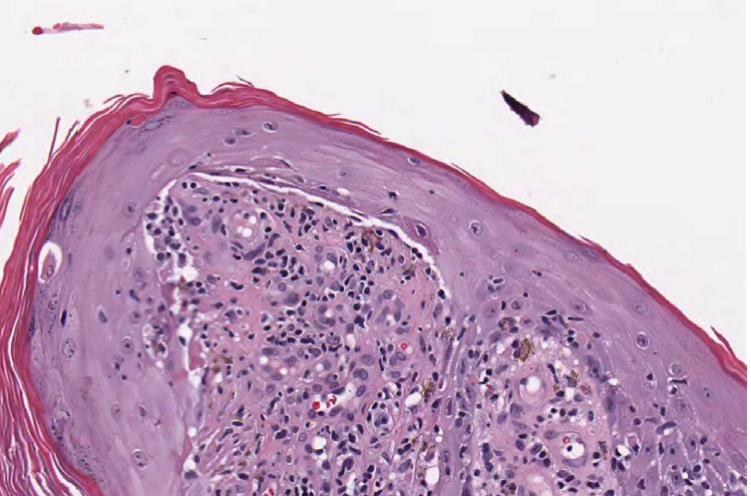
**Case 52, seborrheic keratosis**. Case 52 was a seborrheic keratosis. One study pathologist diagnosed actinic keratosis citing the mild atypia in the epithelium. Consensus was that the correct diagnosis was seborrheic keratosis. The atypia may have been secondary to human papilloma virus (HPV). Figure 5 shows a lower power view of a tissue section.

▪ **Case 52 **was a shave biopsy of skin. A pathologist diagnosed actinic keratosis citing mild atypia in the epithelium (Figures 5 and 6). In committee however, the pathologists agreed that the findings best fit the diagnosis of inflamed seborrheic keratosis with human papilloma virus (HPV) involvement as the atypia was limited to the superficial (not basal) layers, there was a "warty" koilocytotic appearance to the atypical cells and there was a lack of appreciable solar elastosis.

The root cause of these type 1 discrepancies seemed to be lack of experience signing out dermatopathology cases and there was very good evidence that image quality did not play a role in the errors.

Type 2 discrepancies: In these situations, a pathologist did not look at (and therefore did not report on) an area containing a clear, well-imaged lesion.

**Figure 7:**
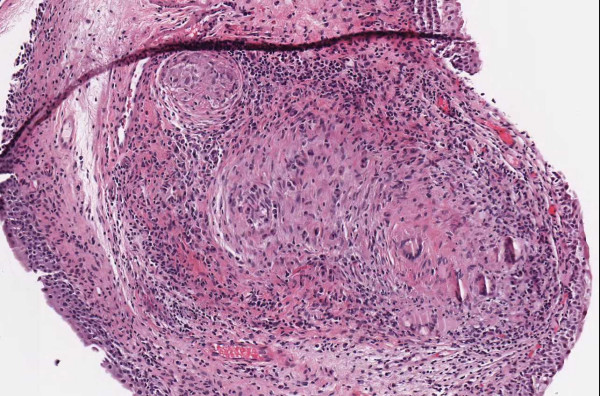
**Case 38, granulomatous inflammation**. A clear granuloma missed by two pathologists as they steadfastly searched for neoplasia in the epithelium

There were three such discrepancies, two in the same case:

▪ **Case 38 **was a two part bladder biopsy that showed chronic inflammation and no neoplasia. There were three levels cut on each part. In part 1, two of the three pathologists missed a focal area suspicious for non-necrotic granulomatous inflammation clearly visible on the WSI image. In part 2, in the last level, there was definite granuloma by WSI (Figure 7). Two of the three pathologists did not see (or report on) these lesions as they were conscientiously seeking neoplasia in the epithelium.

▪ In **Case 55**, a skin biopsy, one of the pathologists diagnosed a junctional nevus and overlooked a small dermal component.

The root cause of these discrepancies seemed to be that the pathologist did not look at and therefore did not "see" the lesion. There was little evidence that image quality was at fault as once the lesion was pointed out there was no question or debate of its presence or significance. However, it is possible that the relative novelty of examining cases on the monitor (and navigating with a mouse) may have contributed to these errors.

Type 3 discrepancies: In these situations, the pathologists agreed on what they saw but disagreed on how to report it. These cases involved significant, legitimate diagnostic uncertainties and tended to reflect differences in judgments on grading in borderline cases or aggressiveness in interpreting superficial invasion in the context of intense inflammation. To qualify as a type 3 discrepancy, there needed to be convincing evidence that image quality did not contribute to the differences in judgment.

**Figure 8:**
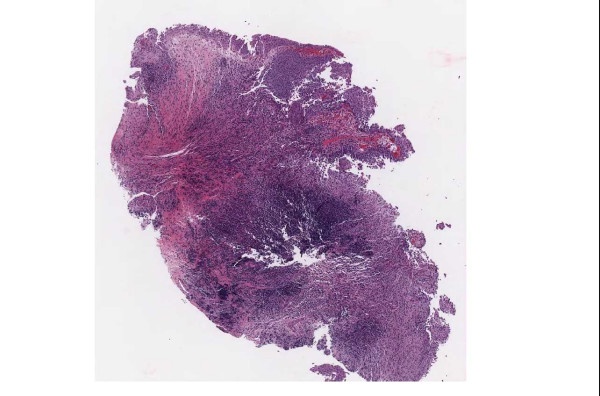
**Case 40, bladder biopsy with inflammation**. Intense inflammation made evaluation of superficial invasion difficult, even under the microscope. Figures 9 and 10 show higher power views.

**Figure 9:**
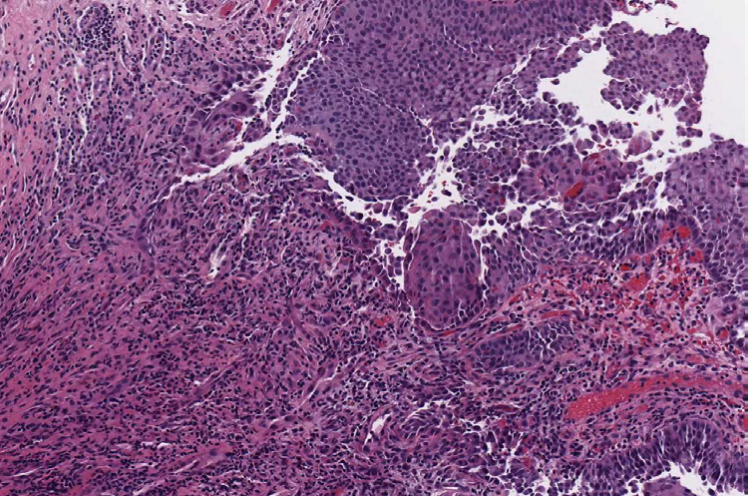
**Case 40, bladder biopsy with inflammation**. Intense inflammation made evaluation of superficial invasion difficult, even under the microscope. Much of the tumor appeared low grade. Figure 8 shows a lower power view.

There were three such discrepancies:

▪ In **Case 40**, a bladder biopsy positive for urothelial neoplasia, one discrepancy involved potential superficial invasion of the lamia propria in the context of extensive inflammation (Figures 8 and 9). The study pathologists reported different opinions. One called it in situ disease, one called it superficial invasion, and one hedged – reporting that "definitive invasion could not be identified" (Appendix). During consensus committee discussion and examination of the images, it was agreed that, although there was legitimate suspicion of invasion, no definitive foci of invasion could be identified. Significantly, the original sign-out pathologist (using the microscope) had reached the same conclusion on H&E slides, and the study pathologists were not swayed from their WSI-based opinions when they later examining the case under a microscope. This case is discussed further below.

**Figure 10:**
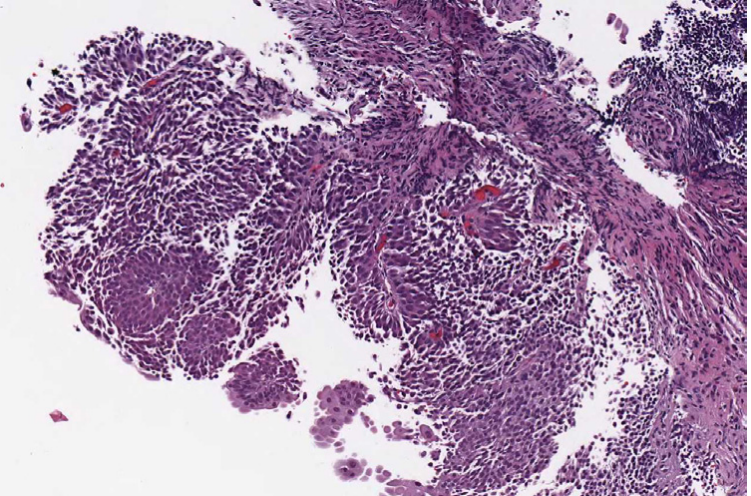
**Case 40, bladder biopsy with inflammation**. In several foci, the tumor cells seemed to loose polarity and had a high nuclear/cytoplasmic ration. Figure 8 shows a lower power view.

▪ A second discrepancy in **Case 40**, involved low versus high-grade neoplasia. This was a difficult call even under the microscope (Appendix). In many areas, the lesion appeared low grade – it did not show marked nuclear polymorphism or increased nuclear size (Figure 9). However there were foci in which the cells were crowded and had a high nuclear/cytoplasmic ratio (Figure 10). After discussion, it was decided that the tumor should be considered a high-grade neoplasm.

▪ **Case 45**, a transurethral resection of a bladder tumor, had a similar scenario. In this case the uncertainty involved potential invasion of the detrusor (Appendix).

**Figure 11:**
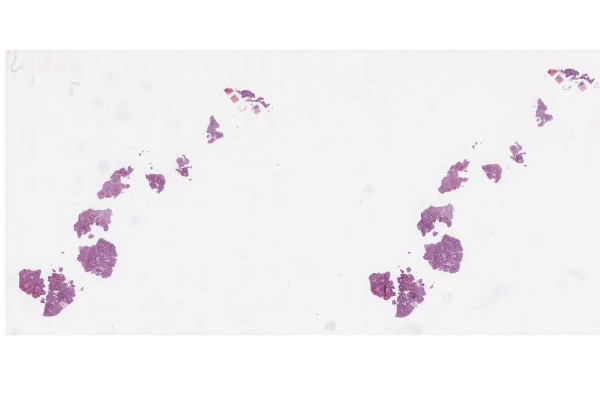
**Case 37, bladder biopsy with urothelial carcinoma**. An overview of the specimen, the diagnostic question was high grade versus low grade. Figures 12 and 13 show higher powers

**Figure 12:**
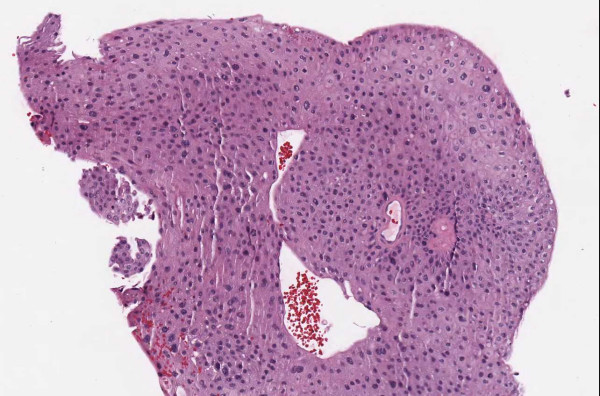
**Case 37, bladder biopsy with urothelial carcinoma**. Medium view of a tissue fragment, the diagnostic question was high grade versus low grade. Figures 11 and 13 show additional views

**Figure 13:**
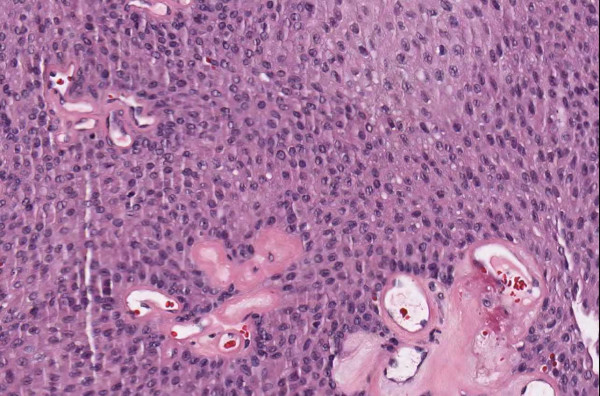
**Case 37, bladder biopsy with urothelial carcinoma**. High power view of a tissue fragment, the diagnostic question was high grade versus low grade. Figures 11 and 12 show additional views

▪ **Case 37**, a bladder biopsy, the question involved low-grade versus high-grade urothelial cancer and image quality seemed to be good. Two study pathologists called for high-grade and one for low-grade. It was a judgment case and consensus opinion was for high-grade. (Figures 11, 12 and 13).

In Case 40 and 45 discrepancies appeared to arise from personal reporting styles and aggressiveness when faced with a legitimately difficult case. WSI quality did not appear to factor into the discrepancies. The root cause of type 3 discrepancies was that these were difficult cases with legitimate uncertainty requiring pathologist judgment. Significantly, the consensus WSI diagnosis turned out to be consistent with the microscope-based, sign-out report in all cases (vide infra).

Type 4 discrepancies: In these cases, image quality was implicated (at least partially) in the disagreements between pathologists.

**Figure 14:**
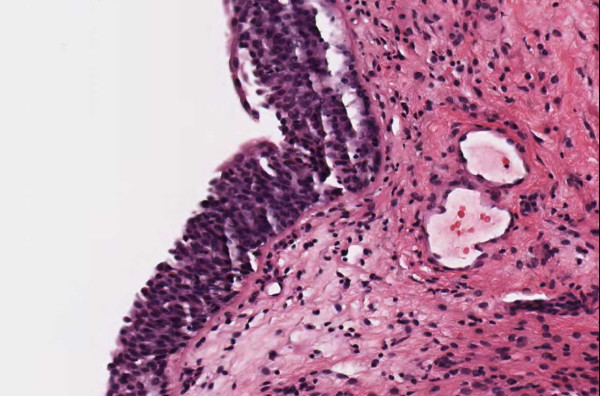
**Case 38, bladder biopsy with urothelial carcinoma**. High power view of the epithelium. The field is slightly out of focus, the epithelial cells are dark and one cannot appreciate and detail in their nuclei. Umbrella cells are clearly visible the upper part of image, but are not present in the center and lower areas. Without umbrella cells, it might be difficult to distinguish between a benign or dysplastic epithelium. There are at least to image problems present: focus and dynamic range (lack of detail in the dark nuclei). Though nuclei can be dark for reasons not related to images (i.e. histologic staining), in this case better nuclear detail was appreciated under the microscope.

There were three such instances:

▪ In **Case 38**, a bladder biopsy, one pathologist reported a focus of high-grade dysplasia while the others reported benign epithelium. Consensus was that the area in question was "suspicious but not convincing of dysplasia" by WSI. The area had epithelial cells with large, dark nuclei and no umbrella cells present; however, similar morphology was seen in other areas covered by umbrella cells (Figure 14). Much of the epithelium seemed slightly out of focus. Traditional microscopic examination (done later) seemed to expose more nuclear detail and made it easier to determine that the epithelium was not dysplastic.

**Figure 15:**
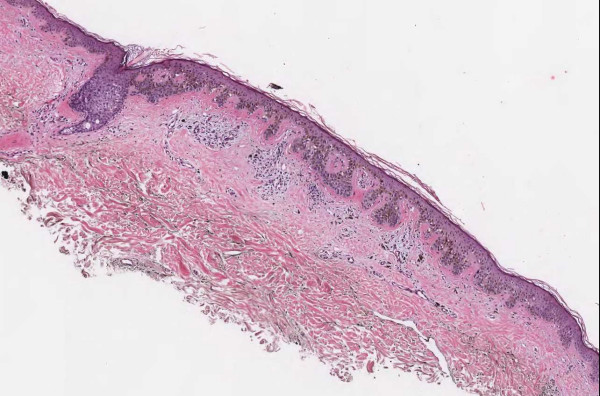
**Case 50, dysplastic nevus**. Less than perfect imaging contributed to confusion in this case. While all pathologists felt that the lesion was a dysplastic nevus, two pathologists could not completely rule out melanoma on H&E stains.

**Figure 16:**
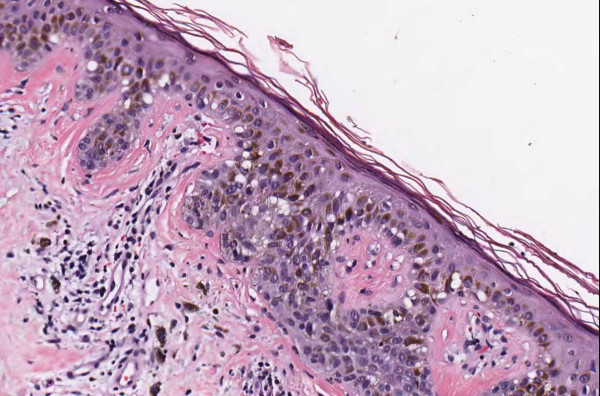
**Case 50, dysplastic nevus**. Less than perfect imaging contributed to confusion in the case. In this field, numerous epithelial melanocytes caused concern for one pathologists that that was magnified because foci were slightly out of focus (i.e. upper left corner).

**Figure 17:**
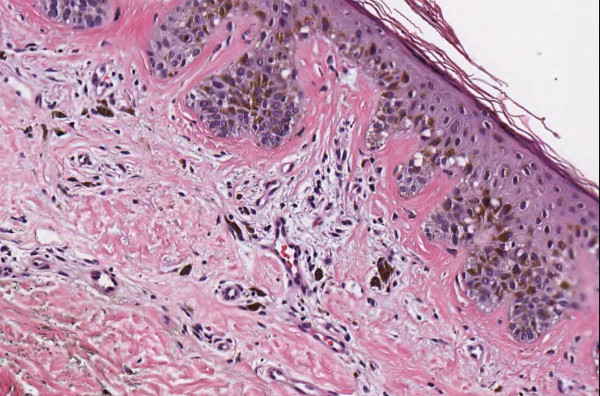
**Case 50, dysplastic nevus**. Less than perfect imaging contributed to confusion in the case. This field shows dermal melanocytes that were difficult to interpret. The pathologist requested immunoperoxidase stains to evaluate the lesion further.

▪ In **Case 50**, a two part skin biopsy, the first part was a basal cell carcinoma and caused no disagreement. In the second part, however, one pathologist (a board certified dermatopathologist) reported a dysplastic nevus (Figure 15) while the other two pathologists reported "atypical melanocytic lesion" and wrote long comments (Appendix). While favoring dysplastic nevus, one was concerned by "areas where single melanocytes are increased in number" (Figure 16) while the other was worried about "atypical melanocytes in the dermis". In consensus, it was agreed that dysplastic nevus was the appropriate diagnosis. This was a legitimately difficult case, but because small areas of the epithelium were out of focus (for example, the upper right in Figure 16) and because dermal melanocytes were difficult to interpret (Figure 17), it was considered a type 4 discrepancy.

**Figure 18:**
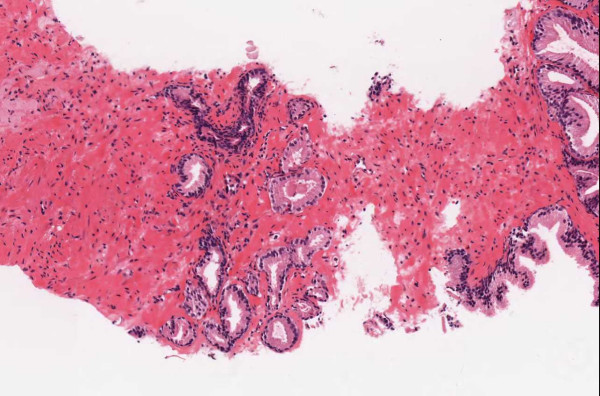
**Case 57, prostate biopsy**. The study pathologists disagreed about this focus. Opinions ranged from benign to suspicious to outright cancer. Special stains were non-contributory. After discussion, it was agreed that cancer could not be definitively diagnosed. This turned out to be consistent with the signed report. The area is slightly out of focus. Figure 19 shows a higher power view.

**Figure 19:**
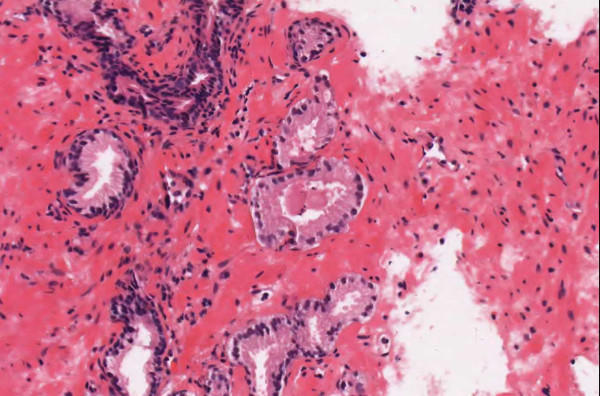
**Case 57, prostate biopsy**. The study pathologists disagreed about this focus. Opinions ranged from benign to suspicious to outright cancer. Special stains were non-contributory. After discussion, it was agreed that cancer could not be definitively diagnosed. This turned out to be consistent with the signed report. The area is slightly out of focus. Figure 18 shows a lower power view.

▪ **Case 57 **was a prostate biopsy. Part 1 had carcinoma and engendered no disagreement. Part 2, however, contained a focus of atypical glands. One pathologist called it "suspicious for carcinoma" and one called it carcinoma outright. Special stains were non-contributory. After significant discussion, it was agreed that carcinoma could not be definitively diagnosed in the image. The field (Figures 18 and 19) was slightly out of focus so image quality was a confounding factor. However, examination under the microscope (in the second part of the study) did not appear to make the decision any easier.

Image quality is implicated (at least partially) in three type 4 discrepancies (Case 38, 50 and 57). However, it is important to note that in all these cases, WSI-based consensus turned out to be in agreement with the (microscope-based) original sign-out report (vide infra). Therefore, even though image quality may be implicated in these discrepancies between pathologists and may have caused confusion, there was enough image information available for a correct diagnosis (vide infra, Discussion).

### Concordance between the WSI consensus diagnosis and the original, microscope-based sign-out report

After examining the individual WSI-based reports during the consensus committee meeting the pathologists agreed on a WSI consensus diagnosis for each case. The WSI consensus diagnosis was written down and then the pathologists were presented, for the first time, with the original, microscope-based, signed-out diagnosis for each case. These data are available in the Appendix.

All 25 cases and 31 parts showed agreement between the WSI consensus diagnosis and the original, microscope-based, sign-out report. Had this been a surgical pathology quality assurance exercise, the study pathologists would not have called a single, even mild, disagreement. This was a remarkable and unexpected finding.

In 21 of the 25 cases, there was line-by-line concordance and these cases were considered in "complete" agreement with the original sign-out report. Four cases had minor differences that required discussion prior to a determination of agreement. These are discussed below.

**Figure 20:**
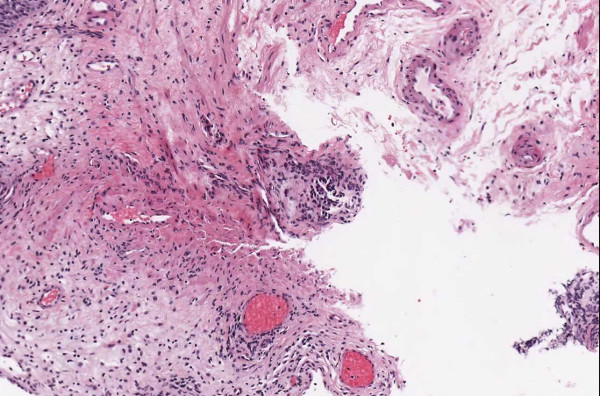
Case 38 Part 1, The signed out report diagnosed "focal submucosal non-necrotizing granulomatous inflammation". The study pathologists (reading from both whole slide images and directly through a microscope) were more comfortable in reporting "focal area suggestive of granulomatous inflammation".

In part one of **Case 38**, a bladder biopsy that showed chronic inflammation and reactive changes, the sign-out report called "focal submucosal non-necrotizing granulomatous inflammation". This was in mild disagreement with the WSI consensus diagnosis, which reported "focal area suggestive of granulomatous inflammation" (Figure 20). After examining the case under the microscope, however, the study pathologists continued to maintain that the findings supporting non-necrotizing granulomatous inflammation were not completely conclusive and that a diagnosis of "focal area suggestive of granulomatous inflammation" was the most appropriate diagnostic line.

The issue appeared to be a matter of pathology judgment and reporting style. If this had been a quality assurance review, in the opinion of the study pathologists, it would not have generated a disagreement, especially since non-necrotizing granulomatous inflammation was definitively called on both imaging and glass slide in the second part of the case (Figure 7). Further, it was clearly not an issue of image quality, as the images were clear and microscopic examination did not cause the study pathologists to change their view. The principle investigator, with the consent of the study pathologists, therefore classified this case as in "basic" agreement.

In **Case 40 **(Figure 8 and 9), a bladder biopsy with high-grade papillary urothelial carcinoma, the issue involved "potential superficial invasion of the lamina propria". It was a very difficult case with marked reactive change. The long diagnostic comment in the original sign-out report confirms the complexity of the case (Appendix) and all of the study pathologists indicted that they would have done an internal consultation before signing it out. Neither the WSI consensus nor the microscope-based original pathologist could document definitive invasion of the lamina propria on H&E stains. The original pathologist, however, ordered and examined an immunohistochemical stain for wide spectrum cytokeratin that confirmed the suspicion of focal, superficial invasion.

The classification of this case was difficult. None of the study pathologists ordered cytokeratin on this case, though all had the opportunity to do so. On the other hand, the WSI consensus diagnosis did not call the case definitively non-invasive; rather, it included the line "no definitive invasion of the lamina propria is identified" and added a comment that "marked reactive changes make it difficult to evaluate this neoplasm". If this had been a quality assurance review, the study pathologists would have had access to the special stains slides used by the original pathologist and would have likely agreed completely with the sign-out report. Because there was no definitive evidence of invasion by H&E and because the WSI consensus diagnosis hedged the issue of superficial invasion the case was classified as "basic" agreement.

**Figure 21:**
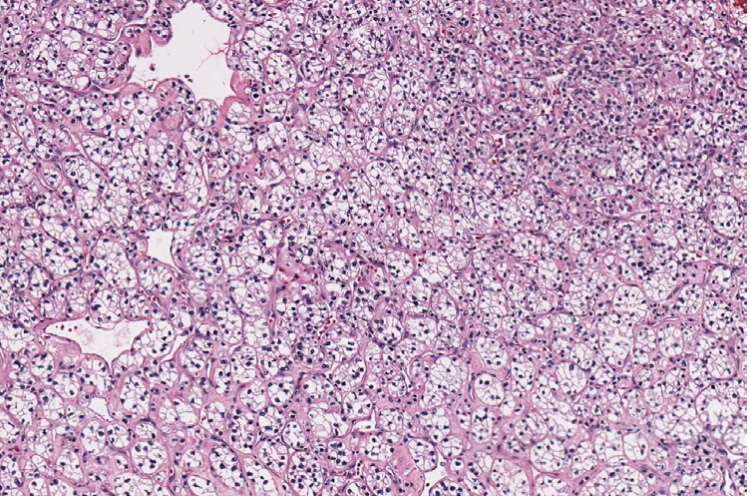
**Case 52, nephrectomy for renal cell carcinoma**. The study pathologists (reading from both whole slide images and directly through a microscope) gave this tumor a Fuhrman's Nuclear Grade of III/IV. The grade in the signed out report is ll/lV.

In **Case 42**, a nephrectomy for renal cell carcinoma, there were two issues (Appendix 1):

1. The consensus WSI diagnosis reported a Fuhrman's Nuclear Grade of III/IV (Figure 21), disagreeing with the sign-out diagnosis of Fuhrman's Grade of II/IV. Interestingly, the individual WSI reports were split on Fuhrman's Grade, two reporting III and one reporting II.

2. The WSI consensus diagnosis of "unremarkable non-neoplastic kidney" conflicted with the original sign-out pathologist report of "non-neoplastic kidney with chronic pylonephritis". Again, in the individual WSI reports, two pathologists called the non-neoplastic kidney "unremarkable" while the third called "mild interstitial nephritis".

It was agreed that these discrepancies would not have caused a disagreement if this had been a QA review. Furthermore, after examining the case under the microscope, the pathologists stood by their WSI consensus diagnoses of Fuhrman's Grade III/IV and unremarkable, uninvolved kidney. The case was therefore classified "basic" agreement. There were no image quality issues evident (Figure 21).

In **Case 45**, a trans-urethral resection of a high-grade urothelial carcinoma, the question involved potential invasion of the detrusor muscle (Appendix). The original sign-out pathologist (using the microscope) had reported that "adequate detrusor muscle is identified without invasion", however, the WSI consensus diagnosis reported "tumor invades the lamina propria" and included the following diagnostic comment: "carcinoma invades at least to the lamina propria, however, due to extensive thermal artifact and tangential sectioning, invasion of the detrusor muscle cannot be completely ruled out." The study pathologists examined the slides under the microscope, and could not be swayed from their opinion. Because both reports concluded the tumor was in the lamia propria and not definitively in the detrusor, the case was classified as one of "basic" agreement. There was no issue of image quality.

### Case evaluation form

In addition to writing a diagnostic report, the study pathologists were asked to complete an evaluation form on each case. Data were collected on image quality, case complexity, diagnostic confidence and system performance.

**Figure 22:**
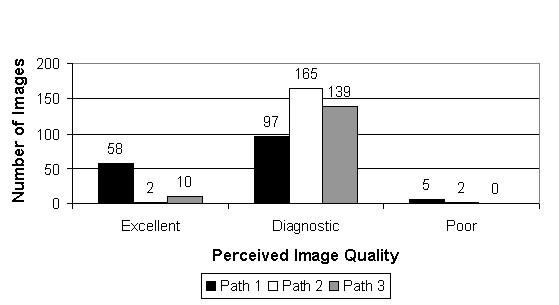
**Perceived image quality**. Each pathologist rated each whole slide image as "Excellent" ("Flawless – superb color and sharpness/focus"), "Diagnostic" ("Minimal distortion – quality is high enough to render a diagnosis") or "Poor" ("Extreme distortion – quality makes diagnosis difficult or impossible"). No whole slide image was rated "poor" by more than one pathologist. Six of seven times, the reason for a "poor" rating was that "key areas were out of focus".

Figure 22 summarizes the pathologists' opinion of image quality. Virtually all of the whole slide images were felt to be either "excellent" ("Flawless – superb color and sharpness/focus") or "diagnostic" ("Minimal distortion – quality is high enough to render a diagnosis"). Only seven images were rated "poor" ("Extreme distortion – quality makes diagnosis difficult or impossible"). Interestingly, no single whole slide image was rated "poor" by more than one pathologist. Six of seven times, the reason for a "poor" rating was that "key areas were out of focus".

**Figure 23:**
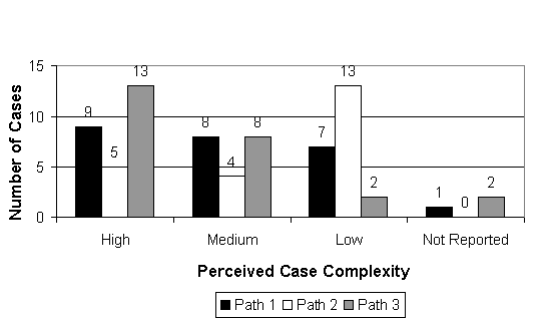
**Perceived case complexity**. Each pathologist rated each case for "Complexity". Complexity involved aspects of diagnostic difficulty, case management (i.e. the need for recuts or special stains), and reporting issues (i.e. the need for a diagnostic comment). The study appeared to include a balanced set of cases with "high", "medium", and "low" complexity.

Figure 23 indicates the pathologists' opinion of the "complexity" of the cases used in the study. "Complexity" is a qualitative measure that involves the diagnostic difficulty, the difficulty of case management (i.e. the need for recuts or special stains) and the difficulty required in expressing a complete and meaningful diagnostic report. The results indicated that the study included a balanced set of cases with "high", "medium" and "low" complexity.

**Figure 24:**
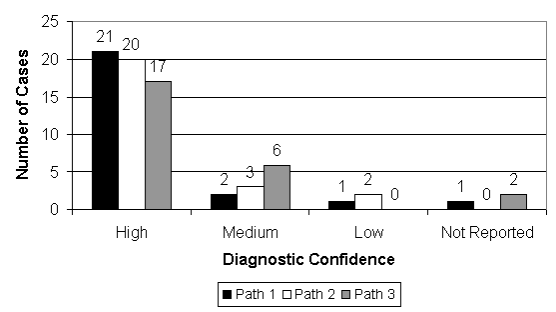
**Diagnostic confidence**. For each case, the pathologists rated their confidence in their diagnosis. Despite the relative novelty of WSI, diagnostic confidence was high. Out of 72 responses, there were only 3 instances of "low" diagnostic confidence (involving cases 30, 35 and 51). These cases did not involve instances of "poor" image quality and did not result in diagnostic discrepancies between pathologists.

Figure 24 shows the degree of confidence pathologists had in their WSI-based diagnoses. Despite the relative novelty of WSI, diagnostic confidence was high. Out of 72 responses, there were three instances of "low" diagnostic confidence (involving cases 30, 35 and 51). These cases did not involve instances of "poor" image quality and did not turn out to result in diagnostic discrepancies between pathologists.

**Figure 25:**
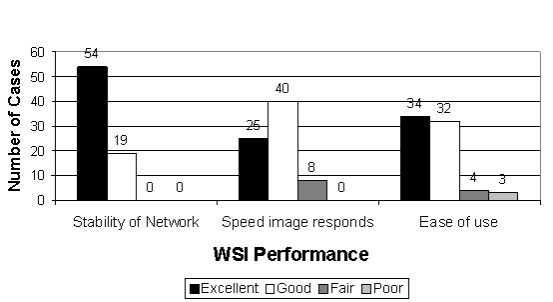
**System performance**. Ratings provided an impression of pathologists' satisfaction with system performance (stability, response times and ease of use). The data is not quantitatively significant, but system performance was felt to be excellent to good in most cases.

Figure 25 gives an impression of the pathologists' satisfaction with the performance of the display system. While the data is by no means quantitatively significant, system performance (stability, response times and ease of use) was felt to be excellent to good in most cases.

**Figure 26:**
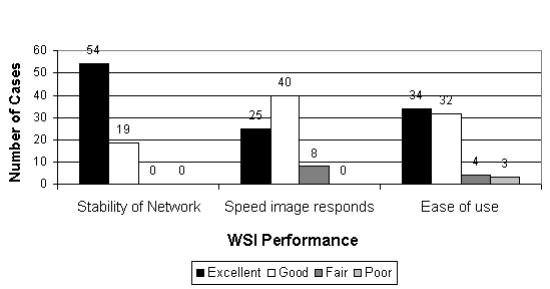
**Time to case completion**. Though useful, this data was very hard to interpret as it included not only "monitor time" but also time required to write the report, order stains and, in some cases, work directly with histology lab to identify and orient blocks.

Figure 26 displays the "time to complete" each case. Though useful, this data is very hard to interpret as it includes not only "monitor time" but also time required to write the report, order stains and, in some cases, work directly with the histology lab to identify and orient blocks.

In general, data from the evaluation forms indicated that the study included a reasonable spectrum of complexity, image quality was considered good, and diagnostic confidence was high. It also indicated that, though system performance (stability, response times and ease of use) was generally good, sign-out times seemed to be longer with current WSI technology than with traditional microscopic examination.

### Focus group de-briefing

The post study focus group exercise was designed to allow study pathologists to voice opinions on issues that may not be directly related to the study's goals. Four main themes were identified: 1) image quality, 2) monitor quality, 3) viewer functionality, and 4) the effect of experience.

Image quality was felt to be good but not perfect. While the great majority of images were considered diagnostic (vide supra), virtually all slides had some areas that were slightly out of focus. These were often described as areas of "softness". One pathologist stated, "...the images were better than those from a resident's microscope but not as good as those through an attending's microscope". These comments seemed consistent with the results from the evaluation form and success of the main primary diagnosis section of the study.

All three pathologists felt that the monitor was a major factor in perceived image quality. Though pathologists disagreed on which type of monitor was better (LED versus CRT), there was strong agreement that, within a given type, higher, more expensive monitors provided much better image quality (and diagnostic confidence). Interestingly, all pathologists agreed that at a given monitor resolution, larger monitors provided better images that were easier to read. One pathologist stated, "I went from a 15 to a 20 inch monitor... A larger monitor made things a lot more comfortable and intuitive for me". Another said, " [The quality of] whole slide images varies with the quality of monitor used as [the quality of] glass slide images varies with the quality of microscope used". These findings are important as the manufactures of WSI equipment are largely focused on improving the quality of image capture, not image display.

Viewer functionality was an area of extensive discussion. The viewer used in the study was similar to others used in the industry. It used a single monitor, numerous mouse-activated buttons and controls, and a small "navigator" or "thumbnail" window to orient the user to the slide. Clinical information and image metadata were displayed in a separate window, competing with the main image for "monitor real estate". The viewer also had no capability to view "thumbnails" of all slides from a given case at the same time (a "slide tray" functionality). The study pathologists found several problems with the design. While there was much debate over details, several important themes emerged:

• The pathologists were irritated that they needed to take their eyes off of the image to move a curser to a button each time they wanted to change magnification, etc. They all expressed the need for more intuitive, less invasive controls.

• The pathologists felt that panning or scanning at low or medium magnification was clumsy and slow. This was not an issue of network/server response time as response times were quite fast. Essentially, the "thumbnail" was too small and did not have enough resolution for diagnostic use and the field of view in the main image window was too small (vide infra). This forced the pathologists to scan through many, relatively low or mid-power fields to evaluate a specimen such as a TURP and may have contributed to the "type 2" errors seen in cases 38 and 55.

• The lack of a thumbnail "slide box" made it difficult to evaluate a case prior to detailed examination of each slide.

Discussion of viewer functionality and monitors led to a discussion of the ideal viewing work-station. According to the study pathologists, the ideal system would have three monitors: a small monitor to display clinical data and image metadata; a large, high-resolution monitor with the same aspect ratio as the slide, mounted horizontally for navigation and to provide an uninterrupted, low to middle resolution image of the entire slide; and a high quality, 17–21 inch monitor for high-resolution evaluation of specific areas. The mouse and keyboard were felt to be less than optimal for controlling the system.

Finally, it was agreed that pathologists seemed to get better at WSI interpretation with experience. Comments included: "...it gets better as you look at a lot of WSI. You learn to maneuver, adapt and acquire short cuts." "WSI requires one to get to a comfort zone after you learn the details of the process." And, "As I see more [images] I get pickier; I can pick up small focusing flaws and compression artifacts." There seems to be a learning curve. It is not steep, but it did exist.

## Discussion

The main goal of this study was to evaluate the clinical utility of current automated, high-speed, high-resolution, WSI robots by evaluating the ability of pathologists to sign-out complex cases exclusively through digital slides created by those devices. The study had two related components. In the first, three study pathologists independently "signed-out" 25 genitourinary pathology and dermatopathology cases exclusively with digital slides. Each pathologist composed a complete final diagnosis including diagnostic comments when appropriate. The work was done through our institution's LIS workflow and the pathologists could order special stains and recuts. The reports from the different pathologists were then compared, discrepancies examined, and a consensus diagnosis was rendered. In the second part of the study, the WSI consensus diagnosis was compared with the microscope-based, sign-out report of the original case pathologist.

The comparison of the WSI consensus diagnosis with the original microscope-based, sign-out report had a remarkable and unexpected result. There was virtually complete diagnostic agreement between the WSI-based report and the microscope-based report for all 31 surgical parts of the 25 cases examined. The results seem to indicate that the image information contained in current whole slide images is sufficient for pathologists to make reliable diagnostic decisions and complex diagnostic reports. This should be very encouraging to the WSI industry; however, these results should not be interpreted as implying that automated, high-speed whole slide images are "as good as" the microscope. In fact, every whole slide image in the study had a least some areas of imperfect focus, and some had what appeared to be compression artifact. In other words, what seems to be impressive diagnostic capability reported in this study should not be construed to mean the images obtained in the study were perfect, or that they were as good as the images formed on the retinal by a well-aligned microscope.

The comparison between the individual WSI-based reports of the study pathologists was also encouraging. In 17 of the 25 cases (21 of 31 specimens/parts) the reports of the study pathologists showed complete agreement. Of the 12 documented discrepancies (in 10 specimens/parts), 9 did not appear to involve image quality issues but three did (vide supra, Results). Looking at this in another way, there were three pathologists who independently rendered extensive diagnoses on 31 parts (93 diagnostic events). Of these 93 events, image quality and/or interpretation issues were implicated, at least partially, in 3 (in cases 38, 50 & 57). It is these cases that are the most interesting and informative. The majority of these discrepancies involved largely cytologic decisions along the continuum of benign, atypical, suspicious to malignant, and while the WSI consensus arrived at the correct diagnosis, the pathologists agreed that examination under the microscope made the decision much easier.

Examining these discrepancies carefully, one can identify some degree of poor focus in the areas in question resulting in blurring of nuclear details. In some cases this may be exacerbated by compression artifact and some degree of limited dynamic range (causing dark area, like nuclei, to appear darker than under the microscope). It is very likely that these effects interact with each other (i.e. poor focus blurs features that may be further eroded by compression). While compression artifact can be improved by changing compression parameters, methods for focus and dynamic range appear to require further development for automated, high-speed WSI systems to be used confidently in clinical primary diagnosis. We are examining these features carefully with controlled experiments and will report our findings in a separate publication.

**Figure 27:**
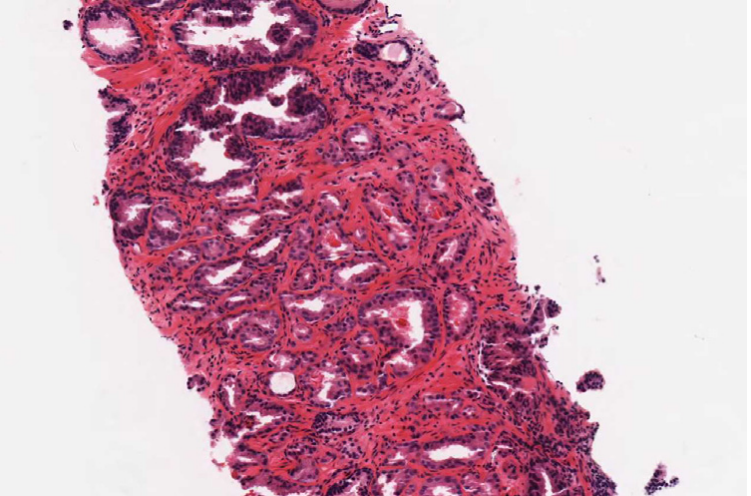
**Case 57, poorly imaged area of adenocarcinoma**. This image shows several image problems. The field was poorly focused and the nuclei appear smeared. Because the rest of the case had reasonably good imaging (including other levels of the same lesion), the pathologists could easily read around the artifact and arrived at the correct diagnosis.

It is important to note that these effects, though they were identified as causes of discrepancies in a relatively small number of cases and did not cause a single mis-diagnosis in consensus, could, in fact, be seen in the great majority of slide images in this study. Virtually every whole slide image had some areas of limited focus and some had areas of what appeared to be compression artifact. However, pathologists could "read around" these areas to find areas of excellent image quality, and they quickly learned how to interpret these "artifacts". For example, Figure 27 shows an area in a prostate biopsy with poor focus and other artifacts (it is from a whole slide image that had excellent focus over large areas). Given the ability to evaluate the entire slide, the pathologists had no problem identifying this area, correctly, as cancer.

The study was designed to measure a very specific thing: the ability of pathology to sign-out completed cases using currently available commercial WSI technology. This study was conducted using an Aperio "T2" high-speed, high-resolution, automated WSI robot. We have several different WSI devices in our lab, from a number of vendors. We chose the Aperio for this study because Aperio is the market leader today, making it a good example of a "typical" modern, WSI device. It is our experience that the results of this study, the good and the bad, are to some degree representative of all automated, high-speed devices we have seen, and they spring from the same limitations that all high-speed devices share – the challenges of focus, speed, and compression.

The study involved a small number of pathologists and a relatively small number of cases. Its interpretation required the comparison of complex, free text diagnostic reports. These limitations make it difficult to generate meaningful statistics and should be taken into account when evaluating the conclusions presented. It should also be noted that the design of a study can significantly impact its results. In the current evaluation, we did a line-by-line comparison of the written reports of study pathologists using WSI, then had the same pathologists develop a written consensus WSI report. We then compared that report, line by line, with the original microscope-based, sign-out clinical report. We felt that this approach allowed us to examine the variation between study pathologists, and, at the same time, to compare a consensus opinion against a clinical "gold standard". Other approaches were possible. For example, we could have randomly assigned pathologists to a modality (WSI or microscope), compared reports, and changed assignments for every case. Given the range of experience in our pathologists (and the limited number of case), this could have caused significant bias and would not have given us a gold standard report to judge against. Alternatively, we could have had the same pathologists see the same cases with both modalities, separated by a time period long enough for the pathologists to forget their initial impression. However, given the complexity of some of the cases and the need for diagnostic comments, recuts and special studies on a significant number of the cases, we felt that the required latency time period would be prohibitively long, especially given that rapid technical progress of whole slide imaging systems. It is also important to note that, in comparison studies, the way that "agreement" and "disagreement" are judged (for example, line by line comparison of written reports or a simple "benign versus malignant"), clearly affects the reported results. Because of this real effect, we decided to make all of our data available in a supplemental file (Appendix 1) so that readers can better interpret the findings for themselves. In the future, to better assess the capabilities of current WSI technology and to validate our findings we plan to build on this study, including more controlled assessments of conditions that may impact the performance, user attributes and application of WSI across a variety of clinical environments.

## Conclusion

Automated, high-speed, high-resolution WSI is an evolving technology that holds great promise for pathology practice. At least in this study, the results indicated that images produced by current devices have enough image information to allow pathologists to produce accurate, complex, and detailed diagnostic reports, even for difficult and complex cases. It is important, however, to recognize that current images have significant limitations that can cause diagnostic confusion, including areas of sub-optimal focus and artifacts that appear to be related to over-compression and limited dynamic range. These limitations must be kept in mind if WSI is to be used for routine clinical primary diagnosis or other clinical applications such as quality assurance or second opinion collaboration. We are beginning to understand these technologies more clearly and this should allow pathologists to better use, and manufactures to better build, WSI systems in the future.

## Abbreviations

CRT – cathode ray tube

HIPAA – Health Information Portability and Accountability Act

IRB – Institutional Review Board

LED – light emitting diode

LIS – laboratory information system

RAM – random access memory

WSI – whole slide imaging

CAD – computer-aided diagnosis

CCD – charge coupled device

## Competing interests

The authors have no competing interests save for the following:

YY has been paid to provide English to Japanese translation services for Aperio Corporation (and other companies in the whole slide imaging industry). She is a scientific adviser to Aperio (and other companies in the whole slide imaging industry) and has represented them in Japan.

JRG once was a principle and chief technology officer for Interscope Technologies, a producer of WSI robots and competitor to Aperio. Interscope was sold to Trestle Corporation (another competitor of Aperio) approximately two years ago. JRG does not retain equity or a financial relationship with Interscope Technologies or Trestle.

DMJ was principle investigator on a clinical evaluation of a whole slide imaging system produced by Trestle, a competitor of Aperio. Trestle Corporation paid for the evaluation.

The University of Pittsburgh Medical Center has purchased whole slide imaging devices from a variety of WSI vendors including Aperio.

## Authors' contributions

JRG organized and managed the study and drafted the manuscript. LA was in charge of data management, led the focus group study, managed IRB issues, and assisted in the manuscript. JH, DMJ and AVP acted as study pathologists and assisted in the design of the study. YY provided imaging resources, advice, and expertise.

## IRB approval statement

The University of Pittsburgh Investigational Review Board reviewed this research study, Number 0501128, and approved it as exempt from Federal Policy for the Protection of Human Research Subjects.

## Pre-publication history

The pre-publication history for this paper can be accessed here:



## Supplementary Material

Additional File 1 **Appendix**. This MSWord Table contains original data including individual pathologist's WSI reports, WSI consensus diagnoses, and original sign-out diagnoses. Readers can use this data to evaluate the claims made in the paper. Click here for file
