# Supplementary material for: Primary histologic diagnosis using automated whole slide imaging: a validation study
Source: BMC Clin Pathol. 2006 Apr 27;6:4. doi: 10.1186/1472-6890-6-4 (PMC1525169; doi:10.1186/1472-6890-6-4)
Supplement: Additional File 1 — Appendix. This MSWord Table contains original data including individual pathologist's WSI reports, WSI consensus diagnoses, and original sign-out diagnoses. Readers can use this data to evaluate the claims made in the paper. [file 1472-6890-6-4-S1.doc]

# Appendix: Raw Data – Pathology Reports and Decisions of the Consensus Committee

For each case in the study, the Appendix includes:

The individual (WSI based) reports of the study pathologists

The decisions of the consensus committee and the thought process behind the decisions

The consensus WSI diagnosis

The diagnosis field from the signed-out (microscope based) pathology report

With this data, it is hoped that readers and reviewers can better evaluate the results and conclusions of the study.

The Appendix is a MS Word document. Each case is on a separate page separated by page breaks. Each page is labeled with a header. We recommend on view the document in “Print Layout View”. In “Print Layout View” each case will be listed in a window along the left margin and one can easily ‘click’ between cases.

The data for each case is presented in a template (a table). This template is discussed in detail on the next page.

# Case Data Documentation Template

This is the basic document used to manage data in the study. This template was filled out for each case. The workflow begins in the middle of the form, where the WSI based reports (diagnostic lines) of the individual study pathologist are entered. The study pathologists (and the principle investigator (also a pathologist) then formed a committee to compare the individual reports, examine differences (if any) and form a consensus WSI diagnostic line. The conclusion of the committee and its decision making logic is presented in the bottom box taken from notes made by the principle investigator and the leader of the evaluation team during the consensus meeting.

The consensus committee, after examining and discussing the individual reports (and reviewing the images), developed a WSI consensus diagnosis that was written down by the principle investigator and is presented in the upper left box in the template. After the WSI consensus was written, the consensus committee was presented with the signed out (microscope based) pathology report for the case. This diagnosis field from that report is documented in upper middle box in the template.

The consensus committee then compared the WSI consensus with the Signed-out report to determine the level of agreement. The committee had access to the entire report, all WSI images, all glass slides and a multi-headed scope. The conclusions of the committee (and, in some cases, documentation of the thought process, are documented in the template’s upper right box.

| **CASE TEMPLATE:** | | |
| --- | --- | --- |
| WSI Consensus:  This is the consensus WSI diagnostic field as developed by the study pathologists during the consensus conference and written down by the Principle Investigator before the signed-out report was presented to the group. | Signed Out Pathology Report (Microscope):  This is the diagnostic field from the signed out pathology report, including comments if present. | WSI Consensus versus Signed-out (Microscopic) Diagnosis:  This is the degree of agreement (as determined by the consensus committee) between the consensus WSI diagnosis and the signed-out (microscope based) diagnosis. |
| Reports of the Individual Study Pathologists (WSI): | | |
| Report of Study Pathology 1:  The study pathologist’s report including comments | Report of Study Pathology 2:  The study pathologist’s report including comments | Report of Study Pathology 3:  The study pathologist’s report including comments |
| WSI Consensus Committee Discussion:  Level of agreement between individual WSI based reports as determined by the committee as well as comments describing the thought processes involved in the consensus | | |

# Case 31: Urinary Bladder Biopsy

| **Case 31: Urinary Bladder Biopsy** | | |
| --- | --- | --- |
| WSI Consensus:   - Urothelial carcinoma, high grade - The carcinoma invades the lamina propria - Fragments of detrusor muscle are free of tumor - Angiolymphatic invasion is not identified | Signed Out Pathology Report (Microscope):   - Fragments of invasive urothelial carcinoma, high grade - The carcinoma invades the lamina propria - TNM histopathological grade = G3 - Detrusor muscle is identified without invasion - No evidence of angiolymphatic invasion is identified | WSI Consensus versus Signed-out (Microscopic) Diagnosis:  **Complete Agreement** |
| Reports of the Individual Study Pathologists (WSI): | | |
| Report of Study Pathologist 1:  Urinary Bladder Tumor, Site Not Specified, Transurethral Resection:   - Urothelial carcinoma, high grade - The carcinoma is invasive to lamina propria - Fragments of detrusor muscle are seen in the submitted material | Report of Study Pathologist 2:  Urinary Bladder, Bladder Tumor Site, Transurethral Resection:   - Invasive high grade urothelial carcinoma - Tumor invades the lamina propria - Detrusor muscle fragments free of tumor - Angiolymphatic invasion is not identified | Report of Study Pathologist 3:  Bladder, Urinary, TURBT:   - Invasive, high grade urothelial carcinoma - The carcinoma invades the lamina propria - Muscularis propria is present but not involved by tumor |
| WSI Consensus Committee Discussion:  **General Agreement**  Comment: The decision not to report the absence of angiolymphatic invasion by two of the study pathologists was considered a matter of reporting style and discretion | | |

**Case 32 was removed prior to the beginning of the study because it was a vasectomy specimen.**

# Case 33: Urinary Bladder, Post-Mortem Donor Cystectomy

| **Case 33: Urinary Bladder, Post-Mortem Donor Cystectomy** | | |
| --- | --- | --- |
| WSI Consensus:   - Viable donor bladder - Mild chronic cystitis - No evidence of in situ or invasive neoplasia seen | Signed Out Pathology Report (Microscope):   - Moderate chronic cystitis with reactive changes of the urothelial mucosa - No evidence of neoplasia is present | WSI Consensus versus Signed-out (Microscopic) Diagnosis:  **Complete Agreement** |
| Reports of the Individual Study Pathologists (WSI): | | |
| Report of Study Pathologist 1:  Urinary Bladder, Post-Mortem Donor   - Urothelium lined bladder tissue with mild chronic cystitis. - No evidence of urothelial carcinoma in situ (CIS) or invasive urothelial carcinoma seen. | Report of Study Pathologist 2:  Donor Bladder:   - Viable donor bladder - No neoplasia seen | Report of Study Pathologist 3:  Urinary Bladder (Donor) Cystectomy:   - Benign bladder tissue - No evidence of urothelial carcinoma in situ or invasive urothelial carcinoma seen. |
| WSI Consensus Committee Discussion:  **General Agreement** | | |

**Case 34 was removed prior to the beginning of the study because it was a vasectomy specimen.**

# Case 35: Vasectomy

| **Case 35: Vasectomy, Segments of Right and Left, Bilateral Vasectomy** | | |
| --- | --- | --- |
| WSI Consensus:   - Bilateral Vas Deferens Excision, complete cross sections obtained. | Signed Out Pathology Report (Microscope):   - Complete cross sections of both vasa deferentia, right and left. - No specific abnormality | WSI Consensus versus Signed-out (Microscopic) Diagnosis:  **Complete Agreement** |
| Reports of the Individual Study Pathologists (WSI): | | |
| Report of Study Pathologist 1:  Bilateral Vasectomy:   - Complete Transection noted in both tissue fragments | Report of Study Pathologist 2:  Vas Deferens, Segments of Right and Left, Bilateral Vasectomy:   - Complete cross sections obtained in both fragments | Report of Study Pathologist 3:  Vas Deferens, Segments of Right and Left, Bilateral Vasectomy:   - Segments of Vas Deferens, completely transected |
| WSI Consensus Committee Discussion:  **General Agreement** | | |

# Case 36: Vasectomy

| **Case 36: Vasectomy** | | |
| --- | --- | --- |
| WSI Consensus:   - Bilateral Vas Deferens Excision, complete cross sections obtained. | Signed Out Pathology Report (Microscope):   - Segments of histologically unremarkable vasa deferentia, completely transected, clinically labeled right and left. | WSI Consensus versus Signed-out (Microscopic) Diagnosis:  **Complete Agreement** |
| Reports of the Individual Study Pathologists (WSI): | | |
| Report of Study Pathologist 1:  Bilateral Vas Deferens, Vasectomy:   - Complete Transection | Report of Study Pathologist 2:  Bilateral Vas Deferens, Excision:   - Complete cross sections obtained | Report of Study Pathologist 3:  Vas Deferens, Bilateral:   - Complete cross sections obtained |
| WSI Consensus Committee Discussion:  **General Agreement** | | |

# Case 37: Bladder Tumor, Transurethral Resection

| **Case 37: Bladder Tumor, Transurethral Resection** | | |
| --- | --- | --- |
| WSI Consensus:   - Papillary, non-invasive, high grade urothelial carcinoma, - The tumor does not invade the lamia propria - Detrusor muscle is not identified | Signed Out Pathology Report (Microscope):   - Papillary urothelial carcinoma, high grade - No invasion of lamina propria is identified - Detrusor muscle is not present | WSI Consensus versus Signed-out (Microscopic) Diagnosis:  **Complete Agreement** |
| Reports of the Individual Study Pathologists (WSI): | | |
| Report of Study Pathologist 1:  Bladder Tumor, TURBT:   - Urothelial Carcinoma, Low Grade - The carcinoma is not invasive in the submitted material - No detrusor muscle is seen in the submitted material | Report of Study Pathologist 2:  Bladder Tumor, Transurethral Resection:   - Papillary urothelial carcinoma, high grade - No invasion of the lamina propria - Detrusor muscle is not present in these sections | Report of Study Pathologist 3:  Bladder, “Tumor”, Transurethral Resection:   - Superficial fragments of non-invasive papillary high grade urothelial carcinoma - There is no evidence of lamina propria invasion - Muscularis propria (detrusor muscle) is not present |
| WSI Consensus Committee Discussion:  **Mild Disagreement between study pathologists, Resolved**  Pathologists 2 & 3 persuaded pathologist 1 that there was sufficient nuclear atypia to qualify as a high-grade tumor. This seems to have been a judgment call rather than in issue of image quality. | | |

# Case 38: Urinary Bladder Biopsy

| **Case 38: Urinary Bladder Biopsy** | | |
| --- | --- | --- |
| WSI Consensus:  Part 1: Urinary Bladder Biopsy (Trigone)   - Benign Bladder Tissue with mild cystitis cystica, mild chronic inflammation and reactive changes. - Focal area suggestive of granulomatous inflammation   Part 2: Urinary Bladder Biopsy (Posterior Wall)   - Benign Bladder Tissue with Cystitis Cystica, chronic inflammation and reactive changes - Focal Granulomatous Inflammation | Signed Out Pathology Report (Microscope):  Part 1: Urinary Bladder, Trigone, Cystoscopic Biopsy   - Benign urothelial mucosa and submucosa with chronic cystitis and focal submucosal non-necrotizing granulomatous inflammation. - No Detrusor muscle is identified - No Evidence of Neoplasia   Part 2: Urinary Bladder, Posterior Wall, Cystoscopic Biopsy   - Benign urothelial mucosa, submucosa and Detrusor muscle with cystitis cystica, focal non-necrotizing granulomatous inflammation and reactive urothelial changes - No evidence of neoplasia (see comment)   Comment: The non-necrotizing granulomatous inflammation in a patient with a history of superficial, high-grade urothelial neoplasia suggests prior BCG therapy effect. The current bladder biopsies show no evidence of urothelial neoplasia. | WSI Consensus versus Signed-out (Microscopic) Diagnosis:  **Basic Agreement, See Comment:**  Comment: In part 1, the signed out report definitively called “focal submucosal non-necrotizing granulomatous inflammation”. This was in mild disagreement with WSI consensus (which reported “Focal area suggestive of granulomatous inflammation”). After examining the case under the microscope, the consensus committee still maintained that the findings supporting non-necrotizing granulomatous inflammation were not conclusive that a diagnosis of “focal area suggestive of granulomatous inflammation” was most appropriate.  If this had been a QA of the original diagnosis, the study pathologist felt that this would not be considered a discrepancy as Granulomatous Inflammation was definitively called by both modalities on Part 2 |
| Reports of the Individual Study Pathologists (WSI): | | |
| Report of Study Pathologist 1:  Part 1: Urinary Bladder Biopsy (Trigone)   - Focus of high grade urothelial dysplasia - No invasive neoplasm seen   Part 2: Urinary Bladder Biopsy (Posterior Wall)   - Chronic, moderately active cystitis with areas of cystitis cystica - No neoplasm seen | Report of Study Pathologist 2:  Part 1: Urinary Bladder, Labeled Trigone, Biopsy   - Benign urothelium with mild chronic cystitis - No evidence of malignancy   Part 2: Urinary Bladder, Labeled Posterior Wall, Biopsy   - Cystitis Cystica - No evidence of malignancy | Report of Study Pathologist 3:  Part 1: Urinary Bladder, Trigone, Biopsy   - Benign bladder tissue with cystitis cystica, chronic inflammation and reactive changes - Focal Granulomatous inflammation   Part 2: Urinary Bladder, Posterior Wall, Biopsy   - Benign bladder tissue with cystitis cystica, chronic inflammation, and reactive changes - Focal, non-necrotizing granulomatous inflammation |
| Consensus Committee Discussion:  **Disagreement between study pathologists, Resolved**  Pathologist 1 reported a focus of high grade dysplasia. Consensus was that the area in question was “suspicious but not convincing of dysplasia” by WSI. Later, the area was examined under the microscope. Microscopic examination seemed to expose more nuclear detail and made it easier to decide that the epithelium was not dysplasic.  Study Pathologists 2 & 3 reported cystitis cystica in part 1, while pathologist 1 did not. The pathologists agreed that cystitis cystica was present on WSI examination. The discrepancy was attributed to reporting discretion and/or style.  Study pathologist 3 reported “focal granulomatous inflammation” in parts 1 & 2 while study pathologists 1 &2 did not. Consensus agreed that there was a definite granuloma in part 2. However, in part 1, the consensus was the WSI evidence “suggestive of but definitive for granulomatous inflammation”. There was no issue of image quality in this case; the granulomatous inflammation was clearly evident, pathologists 1 & 2 were to focused on the epithelium and missed it. | | |

# Case 39: Bladder Biopsy

| **Case 39: Bladder Biopsy** |  |  |
| --- | --- | --- |
| WSI Consensus:   - Urothelial Carcinoma, High Grade - The carcinoma invades the detrusor muscle - No evidence of angiolymphatic invasion | Signed Out Pathology Report (Microscope):   - Invasive Carcinoma involving detrusor muscle (see comment)   Comment: Due to the presence of extensive cautery artifact, neither the surface epithelium nor the architectural and cytologic characteristics of the invasive carcinoma could be evaluated. However, the immunohistochemical results confirm the presence of an invasive carcinoma and favor urothelial origin | WSI Consensus versus Signed-out (Microscopic) Diagnosis:  **Complete Agreement**  Comment: While the surface epithelium was not present, each of the study pathologists felt confident in reporting “Urothelial Carcinoma”. |
| Reports of the Individual Study Pathologists (WSI): | | |
| Report of Study Pathologist 1:   - Urothelial carcinoma, high grade - The carcinoma is invading the detrusor muscle in the submitted material | Report of Study Pathologist 2:   - Invasive urothelial carcinoma, high grade - Tumor invades the detrusor muscle - No evidence of angiolymphatic invasion | Report of Study Pathologist 3:   - Invasive, high grade urothelial carcinoma - The carcinoma invades into the detrusar muscle |
| WSI Consensus Committee Discussion:  **General agreement** | | |

# Case 40: Urinary Bladder, Cystoscopic Biopsy

| **Case 40: Urinary Bladder, Cystoscopic Biopsy** | | |
| --- | --- | --- |
| WSI Consensus:   - Papillary urethlial carcinoma, with areas consistent with a high grade neoplasm - No definitive invasion of the lamina propria is identified, see comment - Detrusor muscle in not present   Comment: Marked reactive changes make it difficult to evaluate this neoplasm | Signed Out Pathology Report (Microscope):   - Papillary urothelial carcinoma, high grade (see comment) - The carcinoma shows microinvasion into the lamina propria (see comment) - No detrusor muscle is present   Comment: The neoplastic cells of the papillary urothelial carcinoma do not show marked nuclear pleomorphism or significant increase in nuclear size. However, they have a high nuclear/cytoplasmic ratio, are very crowded and some of them show a signet ring cell morphology. Therefore, we believe that the lesion would be best classified as high-grade papillary urothelial carcinoma. The biopsy is small and shows marked lamina propria inflammation, probably related to the patient’s history of intravesical BCG treatment. Because of the abundance of inflammatory cells, microscopic invasion by urothelial carcinoma into the lamina propria could not be documented based on the initial hematoxylin and eosin (H&E) stained sections alone. Four additional levels stained with H&E and immunohistochemical stain for wide-spectrum cytokeratin were performed and examined. The immunostain for cytokeratin shows few small groups of cells invading into the lamina propria, confirming the initial impression on H&E sections. Detrusor muscle was not present in the biopsy for full assessment of depth of invasion. However, the lesion was described by Dr. x as very small, with no clinical suspicion of detrusor muscle invasion, which correlates with our finding of only minimal lamina propria invasion by urothelial carcinoma. Dr. x was informed by e-mail on 11/21/2003 about the delay in the release of the pathology report caused  by the additional studies we performed. | WSI Consensus versus Signed-out (Microscopic) Diagnosis:  **Basic Agreement, See Comment:**  Comment: Neither the WSI consensus or the original pathologist could not document definitive invasion of the lamina propria on H&E stains. However, the original pathologist ordered and examined an immunohistochemical stain for wide spectrum cytokeratin which confirmed the suspicion of focal, superficial invasion. None of the WSI pathologists ordered cytokeratin on this case.  This case was considered an “agreement” because 1) The WSI and Signout both agreed on the H&E diagnosis and 2) The WSI consensus did not definitively report a non-invasive tumor. |
| Reports of the Individual Study Pathologists (WSI): | | |
| Report of Study Pathologist 1:  Bladder Biopsy:   - Transitional carcinoma in situ - No invasive areas seen - Chronic, marked active cystitis - Reactive urethelium with reactive mucinous metaplasia and foci of squamous metaplasia - No Detrusor muscle identified | Report of Study Pathologist 2:  Urinary Bladder, Biopsy:   - Superficially invasive high grade papillary urothelial carcinoma - Invasion of the lamina propria - Detrusor muscle in not identified in these fragments | Report of Study Pathologist 3:  Urinary Bladder, Biopsy:   - Non-invasive low grade papillary urothelial carcinoma - No definitive invasion is identified - Detrusor muscle is not present |
| WSI Consensus Committee Discussion:  **Disagreement between study pathologists, Resolved**  Comment: This was a very difficult case and all study pathologists would have shown the case around the department. While the individual reports varied markedly in both tumor grade and invasion, the study pathologist came to consensus that:   1. The tumor was high grade, though the high grade phenotype was most apparent in foci. 2. There was no definitive evidence of invasion, however, the markedly reactive background made invasion hard to interpret. | | |

**Case 41 was removed prior to the beginning of the study because it was a vasectomy specimen.**

# Case 42: Kidney, Simple Nephrectomy:

| **Case 42: Kidney, Simple Nephrectomy:** | | |
| --- | --- | --- |
| WSI Consensus:   - Renal cell carcinoma, clear cell type - Fuhrmans’s Nuclear Grade III/IV - Greatest Diameter of tumor = 5.4 cm - Acinar and Solid Growth Pattern - The tumor is confined within the renal capsule - Renal vein invasion is not identified - Angiolymphatic invasion is identified - All surgical margins free of tumor - Non-neoplastic kidney is unremarkable - Adrenal gland is not submitted - TNM Stage: pT1b N0 MX | Signed Out Pathology Report (Microscope):   - Renal cell carcinoma, clear cell type - Fuhrman’s nuclear grade is II/IV - The greatest diameter of the neoplasm is 5.4 cm - The neoplasm is confined within the renal capsule - No invasion of the renal vein is identified - Angiolymphatic invasion is identified - All surgical margins are free of the neoplasm - Non-neoplastic kidney with chronic pylonephritis - Pelvic sinus fat with focal fat necrosis and chronic inflammation - The adrenal gland is not submitted - Pathologic Stage: T1a Nx Mx - Histologic Grade: G2 | WSI Consensus versus Signed-out (Microscopic) Diagnosis:  **Basic Agreement, See Comment**  Comment:   1. The signed out report reported a Fuhrman’s Grade of II/IV, disagreeing with the WSI consensus and 2 of the 3 WSI pathologist’s individual reports. 2. The signed out report reported the non-neoplastic kidney with chronic pylonephritis. Disagreeing with the WSI consensus and 2 of 3 WSI pathologists.   After examining the case under the microscope, the pathologist stood by their WSI diagnoses of Fuhrman’s Grade III/IV and that the uninvolved kidney was unremarkable  It was the opinion of the pathologists and PI that had this been a QA of the signed-out report, the WSI consensus would not have generated a disagreement. |
| Reports of the Individual Study Pathologists (WSI): | | |
| Report of Study Pathologist 1:  Right Kidney, Laparoscopic Nephrectomy:   - Renal cell carcinoma, clear cell type - Fuhrmans’s Nuclear Grade III/IV - Acinar and Solid Growth Pattern - Greatest Diameter of tumor = 5.4 cm - The tumor is confined within the renal capsule - No Invasion of the renal vein is identified - Foci of angiolymphatic invasion are identified - Non-neoplastic kidney is unremarkable - Adrenal Gland is not submitted | Report of Study Pathologist 2:  Right Kidney, Laparoscopic Nephrectomy:   - Renal cell carcinoma, clear cell type - Fuhrmans’s Nuclear Grade II/IV - Lymphatic invasion is identified - Vascular invasion is not identified - Resected margins free of tumor - Uninvolved kidney with mild interstitial nephritis | Report of Study Pathologist 3:  Kidney, Simple Nephrectomy:   - Renal cell carcinoma, clear cell type - Fuhrman’s nuclear grade III/IV - Greatest diameter of tumor = 5.4 cm - Acinar and solid growth pattern - The tumor is confined within the renal capsule - No Invasion of the renal vein is identified - Angiolymphatic invasion identified - All surgical margins free of neoplasm - Non-neoplastic kidney is unremarkable - Adrenal Gland is not submitted - TNM Stage: pT1b N0 MX * |
| WSI Consensus Discussion:  **General Agreement**  Comment: Two pathologists rated nuclear grade as III/IV while the third rated nuclear grade as II/IV. Consensus was reached at III/IV. | | |

# Case 43: Urinary Bladder Biopsy:

| **Case 43: Urinary Bladder Biopsy:** |  |  |
| --- | --- | --- |
| WSI Consensus:   - Urinary Bladder with benign, largely denuded urothelial epithelium - Mild chronic inflammation - Focal giant cell reaction - No evidence of atypia or neoplasia | Signed Out Pathology Report (Microscope):   - Urinary Bladder wall showing superficial mucosal erosion and minute fragment of benign surface urothelial mucosa - Mild chronic cystitis and lamina propria edema - Granuloma formation - No evidence of neoplasia | WSI Consensus versus Sign-out (Microscopic) Diagnosis:  **Complete Agreement** |
| Reports of the Individual Study Pathologists (WSI): | | |
| Report of Study Pathologist 1:  Bladder Biopsies:   - Fragments of deep bladder wall (predominantly muscularis mucosae) with focal granulomata - Neither neoplasia nor active cystitis is seen | Report of Study Pathologist 2:  Urinary Bladder, Excisional Biopsy:   - Denuded urothelium with mild chronic cystitis and stromal giant cell reaction - No evidence of urothelial atypia or neoplasia | Report of Study Pathologist 3:  Urinary Bladder:   - Mostly denuded, benign bladder tissue with focal giant cell reaction |
| WSI Consensus Discussion and Decision:  **General Agreement** | | |

# Case 44: Prostate, Transurethral Resection

| **Case 44: Prostate, Transurethral Resection** | | |
| --- | --- | --- |
| WSI Consensus:   - Benign prostatic hypertrophy with stromal nodules and adenosis. - Chronic, moderately active prostatitis - No evidence of neoplasia | Signed Out Pathology Report (Microscope):   - Benign prostatic tissue with marked stromal and glandular hyperplasia - Mild chronic active prostatitis - No evidence of neoplasia | WSI Consensus versus Sign-out (Microscopic) Diagnosis:  **Complete Agreement** |
| Reports of the Individual Study Pathologists (WSI): | | |
| Report of Study Pathologist 1:  Prostate, Transurethral Resection:   - Benign prostatic hyperplasia with transitional metaplasia. - Two stromal nodules - Chronic, moderately active, prostatitis - Focus of acute prostatitis noted - No evidence of neoplasia | Report of Study Pathologist 2:  Prostate, Transurethral Resection:   - Benign prostatic parenchyma | Report of Study Pathologist 3:  Prostate, Transurethral Resection:   - Benign prostatic hyperplasia with foci of adenosis (see comment)   Comment: Admixed with foci of benign prostatic hyperplasia are areas of crowded with a nodular configuration of growth. Special stains were non-contributory and I favor that these foci represent adenosis, |
| WSI Consensus Committee Discussion:  **General Agreement**  Stomal nodules, moderately active chronic and foci of acute prostatitis as well as foci of adenosis were all present. | | |

# Case 45: Urinary Bladder, Transurethral Resection of Bladder Tumor

| **Case 45: Urinary Bladder, Transurethral Resection of Bladder Tumor** | | |
| --- | --- | --- |
| WSI Consensus:  Urinary Bladder, Transurethral Resection of Bladder Tumor   - Invasive, papillary, high grade Urethelial Carcinoma - Tumor invades the Lamina Propria (see comment) - Angiolymphatic Invasion is Not Identified   Comment: Carcinoma invades at least to the lamina propria, however, due to extensive thermal artifact and tangential sectioning, invasion of the detrusor muscle cannot be completely ruled out. | Signed Out Pathology Report (Microscope):  Urinary Bladder, Site not otherwise specified, Transurethral Resection of Bladder Tumor   - Papillary and Invasive Urethral Carcinoma, High Grade - The Carcinoma Invades the Lamina Propria - Adequate Detrusor Muscle is Identified without Invasion - No Evidence of Angiolymphatic Invasion | WSI Consensus versus Sign-out (Microscopic) Diagnosis:  **Basic Agreement, See Comment**  Comment: After examining the case under the microscope, the study pathologists were not swayed from their WSI consensus that “carcinoma invades at least to the lamina propria, however, due to extensive thermal artifact and tangential sectioning, invasion of the detrusor muscle cannot be completely ruled out.”  If this have been a QA of the signed out report, there would have been no disagreement reported as the WSI consensus does not call for detrusor invasion. |
| Reports of the Individual Study Pathologists (WSI): | | |
| Report of Study Pathologist 1:  Old Clot and Bladder Tumor, TURBT:   - Fragments of high grade urothelial carcinoma, invasive at least to the lamina propria - Separate fragments of muscle (see note)   Note: There are dispersed fragments of muscle that likely represents muscularis propriae; however, due to extensive cautery artifact and necrosis, I cannot be sure that these are not fragments of detrusor. Carcinoma invades the lamina propria, but due to the facts stated above, invasion of detrusor muscle cannot be ruled out completely. | Report of Study Pathologist 2:  Old Clot and Bladder Tumor, TURBT:   - Invasive, papillary high grade urothelial carcinoma - Tumor invaded the lamina propria - No evidence of angiolymphatic invasion - Detrusor muscle fragments free of tumor - Fragments of blood clot | Report of Study Pathologist 3:  Urinary Bladder, TURBT:   - Invasive high grade urothelial carcinoma with extensive necrosis and focal rhabdoid differentiation - Foci suspicious for muscularis propria invasion are identified (see comment)   Comment: Histologic sections reveal multiple distorted fragments of high grade urothelial carcinoma admixed with necrotic debris and fibrin. Although foci suspicious for detrusor muscle invasion are identified, a definite diagnosis is not possible due to extensive thermal artifact and tangential sectioning. |
| WSI Consensus Committee Discussion:  **Mild Disagreement, Resolved**  Comment: While pathologist 2 reported definitively that the Detrusor Muscle was free of tumor, the pathologists agreed that because of cautery artifact and tangential sectioning, a better diagnostic message would be “Carcinoma invades at least to the lamina propria, however, due to extensive thermal artifact and tangential sectioning, invasion of the detrusor muscle cannot be completely ruled out”. | | |

# Case 46: Skin, Right Upper Medial Arm, Excision:

| **Case 46: Skin, Right Upper Medial Arm, Excision:** | | |
| --- | --- | --- |
| WSI Consensus:   - Intra-dermal Nevus - The nevus involves the deep margin | Signed Out Pathology Report (Microscope):   - Intra-dermal melanocytic nevus involving the deep margin | WSI Consensus versus Sign-out (Microscopic) Diagnosis:  **Complete Agreement** |
| Reports of the Individual Study Pathologists (WSI): | | |
| Report of Study Pathologist 1:  Skin, Right Medial Arm, Excision:   - Intra-dermal melanocytic nevus, partially neurotized. See Note:   Note: No significant lymphocytic infiltrate is seen to consider the diagnosis of halo nevus. | Report of Study Pathologist 2:  Skin of Right Upper Medial Arm, Shave Excision:   - Intra-dermal nevus | Report of Study Pathologist 3:  Skin of Right Upper Medial Arm, Shave Biopsy:   - Intra-dermal nevus with features of ancient nevus, incompletely excised |
| WSI Consensus Committee Discussion:  **General Agreement**  Comment: The committee considered mention of deep margin status in an inter-dermal nevus a matter of reporting discretion and style | | |

# Case 47: Skin, Right Infra-Scapular Back, Shave Biopsy:

| **Case 47: Skin, Right Infra-Scapular Back, Shave Biopsy:** | | |
| --- | --- | --- |
| WSI Consensus:   - Basal cell carcinoma - Margins involved | Signed Out Pathology Report (Microscope):   - Basal cell carcinoma | WSI Consensus versus Signed-out (Microscopic) Diagnosis:  **Complete Agreement** |
| Reports of the Individual Study Pathologists (WSI): | | |
| Report of Study Pathologist 1:  Skin, Right Infra-Scapular Back, Shave Biopsy:   - Basal cell carcinoma, nodular and morpheaform type. - The carcinoma involves the biopsy margins (see comment)   Comment: Follow up for any clinical persistence or recurrence of this lesion is recommended due histological poor circumscription of this neoplasm and perineural invasion this neoplasm may pursue a more aggressive course than other types of basal cell carcinoma. | Report of Study Pathologist 2:  Skin, Right Infra-Scapular Back, Shave Biopsy:   - Basal cell carcinoma - Margins involved | Report of Study Pathologist 3:  Skin, Right Infra-Scapular Back, Shave Biopsy:   - Fragments of basal cell carcinoma, mixed type - Incompletely excised |
| WSI Consensus Committee Discussion:  **General Agreement** | | |

# Case 48: Skin, Flank/Groin Area, Punch Biopsy:

| **Case 48: Skin, Flank/Groin Area, Punch Biopsy:** | | |
| --- | --- | --- |
| WSI Consensus:   - Atypical lymphoid infiltrate consistent with an early stage of mycosis fungoides | Signed Out Pathology Report (Microscope):   - Atypical, Epidermotrophic lymphomatoid infiltrate, suggestive of a patch stage of mycosis fungoides (Cutaneous T-Cell lymphoma variant) (see comment)   Comment: There are atypical cells seen in the dermis and epidermis; some nuclei are reniform. There is some lining of the lymphocytes seen along the dermoepidermal junction (aka ‘birds on a wire’); some perinuclear halos are seen. Spongiosis is minimal. | WSI Consensus versus Signed-out (Microscopic) Diagnosis:  **Complete Agreement** |
| Reports of the Individual Study Pathologists (WSI): | | |
| Report of Study Pathologist 1:  Skin, Flank / Groin Area, Punch Biopsy:   - Atypical dermoepidermal lymphoid infiltrate of concern for an early variant of cutaneous T cell lymphoma, mycosis fungoides type (see comment)   Comment: This is a T-cell positive infiltrate, with pronounced epidermotropism and relative loss of CD8 and CD7. No significant number of CD79a positive cells is seen. The infiltrate is mainly CD4 positive, with no significant increase in CD30. The other differential diagnosis is that of advanced small plaque parapsoriasis which according to some authors is an early stage of mycosis fungoides. | Report of Study Pathologist 2:  Skin, Flank/Groin Area, Punch Biopsy:   - Perivascular and bandlike lymphocytic infiltrate consistent with early patch stage of mycosis fungoides | Report of Study Pathologist 3:  Skin, Flank/Groin, Biopsy:   - Atypical lymphoid infiltrate, suspicious for mycosis fungoides (see comment)   Comment: There is a dense lymphoid infiltrate at the dermal/epidermal junction with small to medium size lymphocytes. These lymphocytes are T cells with an expression of CD2, CD3 and CD4. Within this population, a reduction of staining is seen with markers for CD7 and CD8, suggesting a decreased or absent expression of the population. This immunoprofile and the histological appearance is suggestive of mycosis funcoides. |
| WSI Consensus Committee Discussion:  **General Agreement** | | |

# Case 49: Skin, Middle Lower Back, Excisional Biopsy

| **Case 49: Skin, Middle Lower Back, Excisional Biopsy** | | |
| --- | --- | --- |
| WSI Consensus:   - Compound melanocytic nevus with architectural disorder and mild to focally moderate cytologic atypia - Margins are free of nevus - Associated tissue defect consistent with previous biopsy site - No residual melanocytic neoplasm in the vicinity of the defect is observed | Signed Out Pathology Report (Microscope):   - Compound nevus with architectural disorder and mild to moderate cytologic atypia - Slide section margins are free of nevus - Cicatrix and changes consistent with previous biopsy site are seen | WSI Consensus versus Signed-out (Microscopic) Diagnosis:  **Complete Agreement** |
| Reports of the Individual Study Pathologists (WSI): | | |
| Report of Study Pathologist 1:  Skin, Middle Lower Back, Excisional Biopsy:   - Compound melanocytic nevus with architectural disorder and mild to focally moderate cytologic atypia - Margins are free of nevus - Associated tissue defect consistent with previous biopsy site - No residual melanocytic neoplasm in the vicinity of the defect is observed | Report of Study Pathologist 2:  Skin, Middle Lower Back, Re-excision:   - Residual nevus, margins free | Report of Study Pathologist 3:  Skin, Middle Lower Back, Excisional Biopsy:   - Compound melanocytic nevus with architectural disorder and moderate cytologic atypia - The nevus is completely excised |
| WSI Consensus Committee Discussion:  **General Agreement** | | |

#

# Case 50: Skin Biopsies

| **Case 50: Skin Biopsies** | | |
| --- | --- | --- |
| WSI Consensus:  Part 1: Right Lower Medial Eyelid, Shave Biopsy:   - Basal cell carcinoma, nodular type - Incompletely Excised   Part 2: Left Upper Back, Shave Biopsy:   - Compound melanocytic nevus with architectural disorder and mild to focally moderate cytologic atypia (dysplasic nevus) (see comment)   Comment: Margins are free | Signed Out Pathology Report (Microscope):  Part 1: Right Lower Medial Eyelid, Shave Biopsy:   - Basal cell carcinoma, nodular type - Biopsied   Part 2: Left Upper Back, Shave Biopsy:   - Compound melanocytic nevus with architectural disorder and mild cytologic atypia (see comment) - Slide sections are free of nevus   Comment: No unusual features such as pagetoid spread, single cell predominance or confluence are seen | WSI Consensus versus Signed-out (Microscopic) Diagnosis:  **Complete Agreement** |
| Reports of the Individual Study Pathologists (WSI): | | |
| Report of Study Pathologist 1:  Skin, Right Lower Medial Eyelid, Shave Biopsy:   - Basal cell carcinoma, superficial and nodular type - Margins involved   Skin, Left Upper Back, Shave Biopsy:   - Compound melanocytic nevus with architectural disorder and mild to focally moderate cytologic atypia (dysplasic nevus) - Margins are free of nevus | Report of Study Pathologist 2:  Skin, Right Eyelid, Shave Biopsy:   - Basal cell carcinoma, nodular type - Margins involved   Skin, Left Back, Shave Biopsy:   - Atypical melanocytic lesion (see comment)   Comment: In part 2 there is an atypical melanocytic lesion which extends close to the margins. The lesion consists mainly of nested, junctional melanocytes which demonstrate moderate atypia. Architecturally, the lesion is symmetrical and appears benign. However, there are a few areas where single melanocytes are increased in number and a malignant melanoma cannot be completely excluded. Dermatopathology consultation is advised. | Report of Study Pathologist 3:  Skin, Right Lower Medial Eyelid, Shave Biopsy:   - Basal cell carcinoma - Incompletely excised   Skin, Left Upper Back, Shave Biopsy:   - Atypical melanocytic lesion, rule out melanoma (see comment)   Comment: This lesion has features of a dysplastic compound melanocytic nevus. However, there are small nests of atypical melanocytes in the dermis which are worrisome. These may represent a component of invasive melanoma or they may be a component of the compound melanocytic nevus. Stains were non-contributory because they were ordered on the wrong block…. |
| WSI Consensus Committee Discussion:  **General Agreement on Part 1**  **Mild Disagreement on Part 2** | | |

# Case 51: Skin, Lower Abdomen, Mid-line at Waist, Shave Biopsy:

| **Case 51: Skin, Lower Abdomen, Mid-line at Waist, Shave Biopsy:** | | |
| --- | --- | --- |
| WSI Consensus:   - Neurofibroma | Signed Out Pathology Report (Microscope):   - Neurofibroma | WSI Consensus versus Signed-out (Microscopic) Diagnosis:  **Complete Agreement** |
| Reports of the Individual Study Pathologists (WSI): | | |
| Report of Study Pathologist 1:  Skin, Lower Abdomen, Mid-line at Waist, Shave Biopsy:   - Neurofibroma | Report of Study Pathologist 2:  Skin, Lower Abdomen, Mid-line at Waist, Shave Biopsy:   - Neurofibroma | Report of Study Pathologist 3:  Skin, Lower Abdomen, Shave Biopsy:   - Neurofibroma |
| WSI Consensus Committee Discussion:  **General Agreement** | | |

# Case 52: Skin, Right Lower Leg, Shave Biopsy

| **Case 52: Skin, Right Lower Leg, Shave Biopsy** | | |
| --- | --- | --- |
| WSI Consensus:   - Inflammed Seborrheic Keratosis with features of Human Papilloma Virus involvement mimicking Actinic Keratosis | Signed Out Pathology Report (Microscope):   - Seborrheic Keratosis, markedly inflamed | WSI Consensus versus Signed-out (Microscopic) Diagnosis:  **Complete Agreement** |
| Reports of the Individual Study Pathologists (WSI): | | |
| Report of Study Pathologist 1:  Skin, Right Lower Leg, Shave Biopsy:   - Seborrheic keratosis, inflamed, with features of human papilloma virus involvment | Report of Study Pathologist 2:  Skin, Right Lower Leg, Shave Biopsy:   - Inflamed seborrheic keratosis | Report of Study Pathologist 3:  Skin, Right Lower Leg, Shave Biopsy:  Actinic (solar) Keratosis |
| WSI Consensus Committee Discussion:  **Significant Disagreement, Resolved**  Comment: Pathologist 3 diagnosed Actinic Keratosis citing mild atypia in the epithelium. However, the consensus opinion (to which all concurred) was that the findings better fit the diagnosis of Sebarrheic Keratosis as the atypia was limited to the superficial (not basal) layers, there was a “warty” appearance of the superficial layer and there was a lack of appreciable solar elastosis. | | |

# Case 53: Skin, Left Calf, Shave Excision

| **Case 53: Skin, Left Calf, Shave Excision** | | |
| --- | --- | --- |
| WSI Consensus:  Skin, Left Calf, Shave Excision:   - Malignant Melanoma, superficially invasive - Breslow’s Thickness: 0.33 mm - Clark’s Level: II - Superficial Spreading Type, Radial Growth Phase Present - Vertical Growth Phase Not Present - Surface Ulcer Not Present - Lymphoid Response at Base is Non-Brisk - Lymphoid Infiltration of Tumor is Mild/Minimal - Mitotic Rate: 0/10 HPF - Margins are Free of Melanoma - TNM Stage (AJCC 2002) = pT1a Nx Mx | Signed Out Pathology Report (Microscope):  Skin, Left Calf, Shave Excision:   - Malignant Melanoma, mainly in-situ, focally microinvasive - Breslow Thickness is 0.29 mm - Clark’s Level II - Superficial Spreading Type, Radial Growth Phase Present - Vertical Growth Phase Not Present - Surface Ulcer Not Identified - Lymphoid Response at Base is Mild - Margins are Free of Melanoma - TNM Stage (AJCC 2002) = pT1a Nx Mx | WSI Consensus versus Signed-out (Microscopic) Diagnosis:  **Complete Agreement**  Comment: There was a 0.04 mm discrepancy in Breslow depth between the signed out report and the WSI consensus. Concern was expressed that the measurement tools (either the microscope or the WSI system or both) may not be appropriately calibrated. |
| Reports of the Individual Study Pathologists (WSI): | | |
| Report of Study Pathologist 1:  Skin, Left Calf, Shave Excision:   - Malignant Melanoma, 0.37 cm in largest horizontal diameter - Breslow’s Thickness: .329mm - Clark’s Level: II - Superficial Spreading Type, Radial Growth Phase Present - Vertical Growth Phase Not Present - Ulceration is Not Present - Lymphoid Response at Base is Non-Brisk - Minimal Number of Tumor Infiltrating Lymphocytes - Mitotic Rate: 0/10 HPF - No Vascular Invasion - No Perineural Invasion - No Microscopic Satellitosis - Margins are Free of Melanoma - TNM Stage (AJCC 2002) = pT1a Nx Mx | Report of Study Pathologist 2:  Skin of Left Calf, Biopsy Excision:   - Melanoma, Superficially Invasive, Margins Free (see comment) - 0.39 Depth - Clark’s Level: II   Comment: The lesion consists of an asymmetrical, poorly circumscribed lesion with pagetoid spread and horizontal bridging. While the lesion is predominantly in-situ disease, one focal area appears to be invasive. The lesion is completely excised. | Report of Study Pathologist 3:  Skin, Left Calf , Excision:   - Melanoma, in-situ and invasive - Breslow’s Thickness: 0.33 mm - Clark’s Level: II - Surface Ulceration in Not Identified - Lymphoid Response at Base in Non-Brisk - TNM Stage (AJCC 2002) = pT1a Nx Mx |
| WSI Consensus Committee Discussion:  **General Agreement**  Comment: The Breslow depth reported by pathologist 2 is likely in error. | | |

# Case 54: Skin, left upper back, shave excision

| **Case 54: Skin, left upper back, shave excision** | | |
| --- | --- | --- |
| WSI Consensus:   - Compound melanocytic nevus with architectural disorder and mild to focally moderate cytologic atypia (dysplastic or Clark’s nevus). Completely removed. - Intradermal melanocytic nevus, partially neuratized, involving the deep margin | Signed Out Pathology Report (Microscope):   - Compound melanocytic nevus with architectural disorder and mild to moderate cytologic atypia (aka dysplastic/Clark’s nevus). Margins free. - Intradermal melanocytic nevus, neurotized, adjacent, focally involving the deep margins | WSI Consensus versus Signed-out (Microscopic) Diagnosis:  **Complete Agreement** |
| Reports of the Individual Study Pathologists (WSI): | | |
| Report of Study Pathologist 1:  Skin, Left Upper Back, Shave Excision:   - Compound melanocytic nevus with architectural disorder and mild to focally moderate cytologic atypia, completely excised - Adjacent Intradermal melanocytic nevus, partially neuratized, involving deep margin of excision | Report of Study Pathologist 2:  Skin, Left Upper Back, Excision:   - Compound dysplasic (Clark’s) nevus. Margins free. - Intradermal nevus, neuralized - Actinic Keratosis, lateral margins involved | Report of Study Pathologist 3:  Skin, Left Upper Back, Shave Excision:   - Compound melanocytic nevus with architectural disorder and mild cytologic atypia, completely excised |
| WSI Consensus Committee Discussion:  **Mild Disagreement between study pathologists, Resolved**  The pathologists agreed that there was no WSI evidence of an Actinic Keratosis. What appeared to pathologist 2 as an AK was in reality a “funny cut” with no evidence of atypia or solar elastosis. Study pathologist 3 had seen the adjacent neuratized nevus but did not report it in the context of a dysplatic nevus. | | |

# Case 55: Skin Excisional Biopsies

| **Case 55: Skin Excisional Biopsies** | | |
| --- | --- | --- |
| WSI Consensus:  Part 1: Skin, Left Flank, Excision:   - Compound nevus with features of regression - Completely excised   Part 2: Skin, Left Lateral Foot, Shave Excision:   - Lentiginous junctional nevus - Completely excised | Signed Out Pathology Report (Microscope):  Part 1: Skin, Left Flank, Excision:   - Compound melanocytic nevus with areas of regression - The nevus is completely excised   Part 2: Skin, Left Lateral Foot, Shave Excision   - Lentiginous junctional melanocytic nevus with some features of nevi of acral skin (see comment) - Margins are free of nevus   Comment: Some single cell predominance, but no confluence or pagetoid spread is seen. Thank you for consulting us in the care of this patient. | WSI Consensus versus Signed-out (Microscopic) Diagnosis:  **Complete Agreement** |
| Reports of the Individual Study Pathologists (WSI): | | |
| Report of Study Pathologist 1:  Part 1: Skin, Left Flank, Excision:   - Compound Melanocytic Nevus with mild architectural disorder and mild cytologic atypia with areas of regression - Slide sections are free of nevus   Part 2: Left Lateral Foot, Shave Excision:   - Lentiginous junctional melanocytic nevus (see comment) - Slide margins are free of nevus   Comment: There is single cell predominance seen on initial levels, but neither confluence nor upward spread is seen. As this is nevus is in acral location, this can be expected in some nevi of acral skin; in any event, the nevus seems to be excised. | Report of Study Pathologist 2:  Part 1: Skin, Left Flank:   - Junctional nevus overlying moderate dermal pigmentation (see comment)   Part 2: Skin, Left Foot:   - Junctional nevus of acral skin | Report of Study Pathologist 3:  Part 1: Skin, Left Flank, Excision:   - Compound melanocytic nevus with features of regression - Margins are free of nevus   Part 2: Skin, Left Lateral Foot, Shave Biopsy:   - Lentiginous melanocytic nevus - Margins are negative |
| WSI Consensus Committee Discussion:  **Mild Disagreement between study pathologists, Resolved**  Part 1 was a compound nevus, through the dermal component was small. Pathologist 2 simply missed it. There does not appear to be an image quality issue. | | |

# Case 56: Prostate Biopsies

| **Case 56: Prostate Biopsies** | | |
| --- | --- | --- |
| WSI Consensus:  Part 1: Left Needle Core Biopsy   - Benign Prostate Tissue   Part 2: Left Needle Core Biopsy   - Benign Prostate Tissue | Signed Out Pathology Report (Microscope):  Part 1: Prostate, Left Lobe, Needle Biopsies:   - Benign Prostate Tissue   Part 2: Prostate, Right Lobe, Needle Biopsies:   - Benign Prostate Tissue | WSI Consensus versus Signed-out (Microscopic) Diagnosis:  **Complete Agreement** |
| Reports of the Individual Study Pathologists (WSI): | | |
| Report of Study Pathologist 1:  Part 1: Prostate Needle Core Biopsy (Left)   - Benign Prostate Tissue   Part 2: Prostate Needle Core Biopsy (Right)   - Benign Prostate Tissue | Report of Study Pathologist 2:  Part 1: Prostate Needle Core Biopsy (Left)   - Benign prostatic parenchyma, no evidence of malignancy   Part 2: Prostate Needle Core Biopsy (Right)   - Benign prostatic parenchyma, no evidence of malignancy | Report of Study Pathologist 3:  Part 1: Prostate Left, Biopsy   - Focal, High Grade Prostatic Intraepithelial Neoplasia   Part 2: Prostate Right, Biopsy   - Focal, High Grade Prostatic Intraepithelial Neoplasia |
| WSI Consensus Committee Discussion:  **General Agreement**  Comment: The pathologists examined the areas reported by pathologist 3 as “focal, high grade PIN”. They agreed that while there were glands with papillary infoldings and slightly enlarged nuclei, the areas were best described as low grade PIN. | | |

# Case 57: Prostate Biopsies

| **Case 57: Prostate Biopsies** | | |
| --- | --- | --- |
| WSI Consensus:  Part 1: Prostate, Right Lobe, Needle Biopsies:   - Adenocarcinoma, poorly differentiated - Gleason score 4+3=7 - The carcinoma involves 3 of 5 cores - No perineural invasion - No angiolymphatic invasion - High grade PIN   Part 2: Prostate, Left Lobe, Needle Biopsies:   - High grade PIN - No invasive component seen in these cores | Signed Out Pathology Report (Microscope):  Part 1: Prostate, Right, Biopsy:   - Poorly differentiated adenocarcinoma - Gleason score 3+4=7 - The carcinoma is present in 3 of 5 cores - No perineural invasion seen - No angiolymphatic invasion seen   Part 2: Prostate, Right, Biopsy:   - Focal high grade PIN | WSI Consensus versus Signed-out (Microscopic) Diagnosis:  **Complete Agreement**  Comment: In part one, the signed out report does not mention high grade PIN. It is not possible to know if this is a “miss” or a matter of reporting style given the higher diagnosis of high grade carcinoma. Under microscopic examination, the study pathologists confirmed the presence of high grade PIN in part 1. |
| Reports of the Individual Study Pathologists (WSI): | | |
| Report of Study Pathologist 1:  Part 1: Prostate, Right Lobe, Needle Biopsies:   - Adenocarcinoma, poorly differentiated - Gleason score 4+3=7 - The carcinoma involves 3 of 5 cores - No perineural invasion - No angiolymphatic invasion - High grade PIN present   Part 2: Prostate, Left Lobe, Needle Biopsies:   - Multi-focal high grade PIN - No invasive component seen in these cores | Report of Study Pathologist 2:  Part 1: Prostate, Right Lobe, Biopsies:   - Adenocarcinoma, moderately differentiated - Gleason score 3+4=7 - No evidence of perineural invasion - No evidence of angiolymphatic invasion - High grade PIN   Part 2: Prostate, Left Lobe, Needle Biopsies:   - Atypical glands suspicious for adenocarcinoma (see comment) - High grade PIN   Comment: In part 2, there is a group of atypical, crowded glands which may represent a foci of adenocarcinoma. Results from p63, racemase and high molecular keratin stains were inconclusive. | Report of Study Pathologist 3:  Part 1: Prostate, Right Lobe, Needle Biopsy:   - Prostatic Adenocarcinoma - Gleason score 4+3=7, involving 1 of 5 cores - Gleason score 3+3=6, involving 2 of 5 cores - No evidence of perineural invasion - No evidence of angiolymphatic invasion - High grade PIN   Part 2: Prostate, Left Lobe, Needle Biopsy:   - Prostatic Adenocarcinoma - Gleason score 3+3=6, involving 1 of 6 cores - Separate small focus of atypical glands - High grade PIN |
| WSI Consensus Committee Discussion:  **General Agreement in Part 1**  **Disagreement between study pathologists in Part 2**  The diagnosis of adenocarcinoma in Part 2 rested on a small focus of atypical glands. Immunoperoxidase stains were non-contributory. Examining the WSI in conference, Pathologist 1 convinced the others that the evidence did not support carcinoma in Part 2. | | |

# Case 58: Prostate Biopsy

| **Case 58: Prostate Biopsy** | | |
| --- | --- | --- |
| WSI Consensus:  Part 1: Prostate Needle Biopsy (Right)   - Prostatic Adenocarcinoma, Moderately Differentiated, Gleason 3+3=6 - Tumor involves 4 of 4 cores - Perineural invasion is not identified - Angiolymphatic invasion is not identified - High Grade PIN is present   Part 2: Prostate Needle Biopsy (Left)   - Prostatic Adenocarcinoma, Moderately Differentiated, Gleason 3+3=6 - Tumor involves 1 of 4 cores - Perineural invasion is not identified - Angiolymphatic invasion is not identified - High Grade PIN is present | Signed Out Pathology Report (Microscope):  Part 1: Prostate Biopsy, Right Lobe   - Moderately differentiated Adenocarcinoma (predicted Gleason’s score 3+3=6) - Tumor involves 4 of 4 cores   Part 2: Prostate Biopsy, Left Lobe   - Moderately differentiated Adenocarcinoma (predicted Gleason’s score 3+3=6) associated with High Grade Prostatic Intraepithelial Neoplasia - Tumor involves 1 of 4 cores | WSI Consensus versus Signed-out (Microscopic) Diagnosis:  **Complete Agreement**  Comment: The signed out pathology report did not mention (affirmatively or negatively) perineural invasion, angiolymphatic invasion and, in one part (part 1), HGPIN. The committee was convinced that HGPIN was present in part 1 and the discrepancies were a matter of reporting style/discretion in a prostate biopsy positive for cancer. |
| Reports of the Individual Study Pathologists (WSI): | | |
| Report of Study Pathologist 1:  Part 1: Prostate Biopsy, Right   - Prostatic Adenocarcinoma, moderately differentiated, Gleason score 3+3=6 - The carcinoma involves 2 of 4 cores - Perineural invasion is not identified - Angiolymphatic invasion is not identified - Two foci of High Grade Intraepithelial Neoplasia   Part 2: Prostate Biopsy, Left   - Prostatic Adenocarcinoma, moderately differentiated, Gleason score 3+3=6 - The carcinoma involves 1 of 4 cores - Perineural invasion is not identified - Angiolymphatic invasion is not identified - One foci of High Grade Intraepithelial Neoplasia | Report of Study Pathologist 2:  Part 1: Prostate Biopsy, Right Lobe   - Prostatic Adenocarcinoma, moderately differentiated, Gleason score 3+3=6 - The carcinoma involves 4 of 4 cores - Perineural invasion is not identified - Angiolymphatic invasion is not identified   Part 2: Prostate Biopsy, Left Lobe   - Prostatic Adenocarcinoma, moderately differentiated, Gleason score 3+3=6 - The carcinoma involves 1 of 4 cores - Perineural invasion is not identified - Angiolymphatic invasion is not identified | Report of Study Pathologist 3:  Part 1: Prostate Tissue, Right Lobe   - Prostatic Adenocarcinoma, moderately differentiated, Gleason score 3+3=6 - The carcinoma involves 4 of 4 cores - Perineural invasion is not identified   Part 2: Prostate Tissue, Left Lobe   - Prostatic Adenocarcinoma, moderately differentiated, Gleason score 3+3=6 - The carcinoma involves 1 of 4 cores - Perineural invasion is not identified |
| Consensus Committee Discussion:  **General Agreement**  Comment: The pathologists agreed that there was extensive WSI evidence for tumor in all four cores in part 1.  All study pathologists agreed that foci of HGPIN were present in both parts and that angiolymphatic invasion was not present. The decision not to report these findings in a prostate biopsy positive for cancer was considered a matter of reporting style and discretion. | | |
